# Picomolar Inhibition of Plasmepsin V, an Essential Malaria Protease, Achieved Exploiting the Prime Region

**DOI:** 10.1371/journal.pone.0142509

**Published:** 2015-11-13

**Authors:** Luca Gambini, Luca Rizzi, Alessandro Pedretti, Orazio Taglialatela-Scafati, Mario Carucci, Andrea Pancotti, Corinna Galli, Martin Read, Emanuele Giurisato, Sergio Romeo, Ilaria Russo

**Affiliations:** 1 Department of Pharmaceutical Sciences, Università degli Studi di Milano, Milan, Italy; 2 Department of Chemistry of Natural Substances, Faculty of Pharmacy, Università di Napoli "Federico II", Naples, Italy; 3 Department of Experimental Medicine and Biochemical Sciences, Università degli Studi di Perugia, Perugia, Italy; 4 Faculty of Life Sciences, University of Manchester, Manchester, United Kingdom; University of Camerino, ITALY

## Abstract

Malaria is an infectious disease caused by *Plasmodium* parasites. It results in an annual death-toll of ~ 600,000. Resistance to all medications currently in use exists, and novel antimalarial drugs are urgently needed. Plasmepsin V (PmV) is an essential *Plasmodium* protease and a highly promising antimalarial target, which still lacks molecular characterization and drug-like inhibitors. PmV, cleaving the PExEl motif, is the key enzyme for PExEl-secretion, an indispensable parasitic process for virulence and infection. Here, we describe the accessibility of PmV catalytic pockets to inhibitors and propose a novel strategy for PmV inhibition. We also provide molecular and structural data suitable for future drug development. Using high-throughput platforms, we identified a novel scaffold that interferes with PmV *in-vitro* at picomolar ranges (~ 1,000-fold more active than available compounds). Via systematic replacement of P and P' regions, we assayed the physico-chemical requirements for PmV inhibition, achieving an unprecedented IC_50_ of ~20 pM. The hydroxyethylamine moiety, the hydrogen acceptor group in P_2_', the lipophilic groups upstream to P_3_, the arginine and other possible substitutions in position P_3_ proved to be critically important elements in achieving potent inhibition. *In-silico* analyses provided essential QSAR information and model validation. Our inhibitors act ‘on-target’, confirmed by cellular interference of PmV function and biochemical interaction with inhibitors. Our inhibitors are poorly performing against parasite growth, possibly due to poor stability of their peptidic component and trans-membrane permeability. The lowest IC_50_ for parasite growth inhibition was ~ 15μM. Analysis of inhibitor internalization revealed important pharmacokinetic features for PExEl-based molecules. Our work disclosed novel pursuable drug design strategies for highly efficient PmV inhibition highlighting novel molecular elements necessary for picomolar activity against PmV. All the presented data are discussed in respect to human aspartic proteases and previously reported inhibitors, highlighting differences and proposing new strategies for drug development.

## Introduction

Malaria, a major killer among infectious diseases, is caused by parasites of the genus *Plasmodium*, among which falciparum is the deadliest strain. Despite malaria morbidity and mortality having recently decreased to 200 million cases per year and a daily rate of ~ 1,600 deaths (WHO—Malaria Report 2014), the current situation is very precarious. Resistance to all available anti-malarials [1], including artemisinin and its derivatives (among the most effective of drugs), is evident and spreading [2]. New approaches to control malaria are a priority and novel antimalarials are urgently needed.

Protein secretion is an indispensable process through which *Plasmodium* substantially rebuilds invaded host cells: new organelles, metabolic functions, nutrient permeation pathways and surface proteins are needed to support parasite growth and infection (reviewed in [3]). Parasites accomplish these changes by exporting hundreds of proteins into the host cell. Parasite protein export relies on diverse signals and trafficking routes [4]. Among them, a novel targeting motif was discovered in 2004, based on the sequence RxLx(x)^E/Q/D^ [5–7]. This unique motif, named *Plasmodium* Export Element (PExEl) [6], identifies ~300 exported proteins, that comprise the so-called PExEl ‘secretome’ [7]. Numerous PExEl proteins have essential functions or are required for virulence traits, including antigen presentation and cell adhesion [8, 9]. All the available data [10–13] strongly indicate that the PExEl-secretion mechanism is an ideal target for novel anti-malarials that would interfere with both, viability and virulence.

Plasmepsin V (PmV) is an essential key factor in PExEl-secretion, as it controls the sorting of the entire PExEl-secretome [10–16]. PmV is responsible for the recognition and cleavage of the PExEl-motif, both essential events for PExEl-secretion [14, 15, 17]. PmV is highly conserved in all *Plasmodium* species with no detected genetic or functional redundancy. It is a unique aspartic protease, absent in the human host, with a peculiar subdomain composition, specific substrates and cellular role [15, 17]. Therefore, PmV is widely recognized as an ideal target for new antimalarial interventions [10, 11, 14, 15, 17, 18]. Despite its crucial importance and potential as novel drug target, *P*. *falciparum* PmV still lacks complete molecular characterization, tridimensional structure and drug-candidate inhibitors.

PmV is minimally affected by HIV-protease inhibitors and Pepstatin A, a general aspartic protease inhibitor [7, 15]. Very recently statine-like scaffolds were shown to also inhibit PmV at nanomolar concentrations [19]. Here, we describe for the first time a novel molecular scaffold with picomolar inhibitory activity against PmV, resulting in molecules which are 1,000-fold more potent than previously reported [19]. In addition, by establishing a multipronged, high throughput platform for synthesis of compounds and detection of PmV activity, we were able to scan the accessibility of the PmV catalytic site; allowing greater molecular understanding of efficient PmV inhibition. To this end, a fast and efficient synthetic approach was developed in order to generate multiple compounds, which were then used for *in-silico* QSAR analyses. Our work, paralleled by recent work [20] that reached publication while our work was in preparation, is one of the first experimental attempts to understand the structural and functional constraints of PmV inhibition. Our analyses revealed crucially important novel elements for PExEl-cleavage inhibition that will pave the way for the design of PmV inhibitors with high potential for anti-malarial drug application.

## Materials and Methods

### Plasmepsin V purification and kinetic measurements

GFP-tagged Plasmepsin V (PmV) was purified from large batches (5–100 billions cells) of parasite pellets (clone DC6 [15]), harvested by centrifugation after saponin treatment to release the majority of RBC cell cytoplasm content [21]. Saponin was added to culture to a final 0.05%; parasites were recovered via centrifugation at 4°C after 5 min of incubation on ice; parasite pellets were then washed twice in cold PBS and either immediately lysed or stored at -80°C until lysis. Parasites were lysed via three pulses of ultra-sonication of 10 sec duration, in PBS containing 0.5% Triton-X100 (TX-100). After incubation on ice with Protein A sepharose for 30 min (Amersham—GE Healthcare Life Technologies), brownish debris and pre-clearing resin were carefully removed by 1–2 steps of centrifugation at 200 x g for 2 min at 4°C. Cleared lysate was then incubated 1–2 h at 4°C, with constant shaking, with anti-GFP antibody 3E6 (Life Technologies—Invitrogen) bound to Protein A sepharose in the following ratio, 0.2 μg of IgG, bound to 10 μl of packed beads, per billion parasites. Quick washes of the resin were performed either in batches, or over columns, to remove unbound cellular material (four with ice-cold 0.5% TX-100 in PBS and a final one with cold PBS). Resin-bound PmV was stored in PBS at 4°C and used within 2–3 weeks, larger batches had Glycerol added to them, up to 10%, and were stored at -20°C for up to 5 months. Both methodologies preserved PmV activity for the stated periods. Quality and purity of PmV batches were routinely confirmed by protein electrophoresis. Quantification of PmV was performed via densitometry analysis of stained protein SDS-PAGE gels relative to BSA standard curves.

PExEl cleavage activity was monitored by fluorimetric measurement of PExEl cleavage, as previously reported [15]. EDANS emission at 490 nm (336 nm excitation) was monitored in a 96-well plate format on an Infinite 500F (Tecan fluorimeter). PmV activity was performed at 37°C in 50 mM Tris-Maleate, pH 6.5, 50 mM NaCl, 0.05% TX-100, 2 mM DTT. Assays were routinely conducted in triplicate. Bead-bound enzyme was added to the reaction using ‘end-cut’ micropipette tips in order to facilitate sampling the suspension.

Steady-state measurements of PmV substrate hydrolysis were analyzed assuming the widely accepted aspartic protease mechanism, *E* + *S* → *ES* → ET* → *EP* → *E* + *P*, where P is the product of the reaction; S is the substrate; E is the free enzyme; ES, ET* and EP denote the enzyme-substrate, the transition state and enzyme-product adducts, respectively. The measurement of steady-state velocity of product formation, *v* = *d*[*P*]/*dt*, yielded catalytic constants according to the classical Michaelis-Menten equation v = e * k_cat_ *[*S*] / ([*S*] + *K*_*M*_), where e is enzyme concentration. In our experimental conditions the fluorescence of HETQ-EDANS (AnaSpec), the reaction product (P), was quantified by titration. PmV kinetics parameters were calculated using non-linear curve-fitting analyses from SigmaPlot on the basis of measurements with multiple substrate concentrations monitored until the completion of the reaction. Total PmV concentration was derived from densitometry analysis of stained protein gel against BSA standard curve and this was assumed to be the maximum concentration of active enzyme.

### Synthesis method

Chemical synthesis was performed employing a high-standard regime and resulted in molecules with a purity ≥ 95%. A flexible protocol that enabled high throughput exploitation of the chemical pipeline of automatic synthesizers was used. The synthesis of hydroxyethylamine (HEA) analogues and the hydroxyl group of HEA isostere was carried out directly on the solid support using the procedure depicted in S1 Fig. The solid phase synthesis was carried out manually on a 150 μmol scale. As solid support we used either polystyrene Rink-Amide MBHA or polystyrene 2-chlorotritylchloride resin (Novabiochem), in order to obtain carboxyamidate compounds and acid free compounds, respectively. Onto these resins the di- or tripeptide **I** was generated by Fmoc/tBu solid phase peptide synthesis (SPPS) (S1 Fig). Successively, the bromomethylketone of Fmoc-leucine reacted with the free amine group of **I** to give the ketomethylenamine **II**, which, after the protection on the amine function with Boc, was reduced with NaBH_4_ in an epimeric mixture of the HEA analogues (compound **III** in S1 Fig). The general procedures for non-standard SPPS are depicted in S1 Fig. After other SPPS cycles to insert into the sequence one or two amino acid residues, final compound **IV** was cleaved from the resin by using a cocktail of trifluoroacetic acid (TFA) and scavengers obtaining a crude mixture of the two epimers **V** and **VI** (S1 Fig).

The products were purified by preparative RP-HPLC (reverse phase HPLC) on a Waters system equipped with photodiode detector array Waters 2996, using a Sunfire C18 OBD Prep column (19 x 150 mm; 5 μm) and a linear gradient of H_2_O (0.1% TFA)/MeOH (0.1% TFA) from 30 to 80% of MeOH (0.1% TFA) in 32 min at a flow rate of 14 mL/min. The analysis and purity determination of the fractions were evaluated by analytical RP-HPLC on a VWR Hitachi—Elite LaChrom system equipped with Hitachi diode array detector L-2450, using an Eclipse XDB C18 column (4.6 x 150 mm, 5 μm). The collected fractions containing the peptides were lyophilised. HR-Mass (high resolution mass spectrometry) analyses were conducted using a MALDI TOF-TOF *AUTOFLEX III* (Bruker Daltonics).

### NMR

The relative configuration of the HEA moiety of the different stereoisomers was assigned through application of the *J*-based configuration analysis, also known as Murata’s method [22]. This is based on the evaluation of ^3^ *J*_H,H_ and ^2,3^ *J*_CH,_ to assign the relative configuration of adjacent stereogenic carbons in acyclic molecules. ^1^H and ^13^C NMR resonances, as well as ^1^H-^1^H coupling constants, were determined as a result of a detailed analysis of 1D and 2D NMR (COSY, HSQC) spectra. The HEA region of the molecule presented a limited degree of conformational mobility, optimal for the applied methodology. Similarly, heteronuclear (^1^H-^13^C) coupling constants were acquired by means of the 2D NMR spectra HETLOC and Phase Sensitive-HMBC. All spectra were acquired on a Varian Inova 700 MHz spectrometer equipped with a cryoprobe at 27°C. Homonuclear ^1^H connectivities were determined by the COSY experiment; one-bond heteronuclear ^1^H-^13^C connectivities by the HSQC experiment; two- and three-bond ^1^H-^13^C connectivities by gradient-HMBC experiments optimized for a ^2,3^ *J* of 8 Hz. Through-space ^1^H connectivities were elucidated by the use of a ROESY (Rotating frame nuclear Overhauser Effect Spectroscopy) experiment with a mixing time of 500 ms.

### Plasmepsin V inhibition

Inhibitory activities were measured in the presence of a substrate concentration of 3 μM by serial dilution of each compound in triplicates. Inhibitors and fluorogenic peptides were introduced to the assay plate using DMSO (final DMSO concentrations 0.29–1.3% (v/v)). The susceptibility of PmV-assay to DMSO was tested, and yielded an unaltered activity profile up to 2% (v/v). Titration curves of the inhibitors were routinely composed of eleven points from 10 μM to 0.1 pM. PmV concentration was kept in the order of low picomolar (≤10 pM). In order to minimize variations between assays, each enzyme batch was assessed for substrate cleavage K_M_, substrate specificity and susceptibility to **Compound 1**. Analysis of the half inhibitory activity (IC_50_) was performed by sigmoidal interpolation of the starting velocities of the hydrolytic reactions for each condition. Starting velocities were calculated on the initial linear range of the product progression curves. **Compound 1** was included in all measurements in order to standardize multiple assays. Data are presented with error bars indicating the standard deviation of three independent activity curves. K_i_ was calculated on the basis of the equation: *IC*_50_ = *K*_*i*_ (1 + [*S*] / *K*_*M*_), and Δ*G*_*binding*_ (Δ*G*_*b*_) = RT ln(*K*_*i*_), where T is the absolute temperature and R is the gas constant.

### Plasmodium cultures and genetic modifications

Strains comprise 3D7, and 3D7-derived clones: DC6, #3, and HRPII-GFP [15]. Clone **DC6** expresses PmV-GFP (green fluorescent protein) under the regulation of the PmV native promoter and has been previously described [15]. Clone #**3** has been previously described, it carries an episomally-maintained plasmid for the expression of full-length PmV-GFP under the transcription regulation of *Hsp86-5'*, a strong and constitutive promoter [15, 23]. Clone HRPII-GFP expresses *Plasmodium* Histidine Rich Protein II (HRPII), an abundant PExEl-secreted protein fused to GFP [15]. As previously described, this modification is compatible with the natural secretion of the HRPII.

Techniques for parasite culture, 3′-end integrations and their analysis, including fluorescence imaging, parasite synchronization and western blotting, have been described previously [15, 23–27].

To evaluate peptide internalization, *Plasmodium* cultures were incubated with **Compound 36**. Three concentrations of **Compound 36**: 500 nM, 1 μM and 5 μM, were incubated for 6 h; parasites were then washed free of the compound and fixed with 4% paraformaldehyde and 0.0075% glutaraldehyde. Fixed samples were then incubated with FITC-Streptavidin and analysed by microscopy and flow cytometry, in the presence of Hoechst or Topro3 (Life Technologies) as previously described [15, 23, 24]. Immunofluorescence analysis was performed using rabbit antibody anti-BiP (MR4), then detected with Alexa594-antibody anti-rabbit (Life Technologies). Nuclei were detected using 4',6-diamidino-2-phenylindole (DAPI).

Pull-down experiments were performed using parasite cellular lysates of DC6 a clone that constitutively expresses PmV-GFP [15]. The lysate was aliquoted in equal volumes and used for either: [i] immune precipitation via anti-GFP antibodies or [ii] pull-down using streptavidin-agarose conjugated with compound **36**. [i] Portions of the clarified lysates were added to either DMSO, or **Compound 36** in absence or presence of **Compound 1** (final concentration of each inhibitor was 50 μM). An anti-GFP antibody, 3E6, was added with protein A-agarose as above described and incubated 1 h at 4°C, with gentle agitation. The resin was washed 3 times with cold PBS and then boiled in Laemmli sample buffer for later biotin detection in a dot-blot format. [ii] 250 μM **Compound 36** was incubated, with gentle agitation, at 4°C for 30 minutes with prewashed 100 μL streptavidin-agarose slurry (Pierce). The resin was then washed 3-times with cold PBS, divided into 4 aliquots and incubated with buffer, or clarified DC6 lysates, in the presence or absence of 50 μM **Compound 1**. Incubation was carried out for 1 h at 4°C gently maintaining the beads in suspension. Then the resin was washed 3 times with cold PBS and boiled in Laemmli sample buffer for subsequent protein western blot analysis of the pull-down material.

Plasmids were constructed to genetically generate PmV knockdown and to express cytosolic YFP-DD, as a control. DD indicates a destabilization domain derived from FK506 binding protein [28], previously used to generate regulatable protein knock-down in *Plasmodium falciparum* [24]. It was obtained by cloning ~1.4 kb of *PmV 3' ORF* into pIRCTGFP-FKBP [24]. While YFP-DD chimera was cloned into the pIRHsp86Rep vector, as previously described [23], it carries a hDHFR cassette, the Rep20 element and an *Hsp86-5’UTR* driven cassette for episomal expression. For transfections, 160 μl of packed RBCs were transfected by electroporation with ~100 μg of purified vector DNA and then infected with *Plasmodium falciparum* 3D7 schizonts to yield a final 5–12% parasitemia. After 72–90 h, 10 nM WR99210 (kindly provided by Jacobus Pharmaceutical Company, Princeton, NJ) was added to the medium. To select for integration, parasites were cycled twice on/off drug [29]. Clone **G6** expresses PmV-GFP-DD and was isolated by limiting dilution and analysed via Southern Blot employing 1 μg of BsrGI-restricted genomic DNA, as previously described [24].

Protein analyses to detect PmV, HRPII and BiP via stained protein gels and blots, densitometry, antibody detection or HRP-conjugated Streptavidin, were conducted as previously described [15, 23, 24].

### Flow cytometry and growth analysis

Growth impairment of parasites in culture was tested as follows. Asynchronous erythrocyte cultures of *Plasmodium falciparum* strain 3D7 were initiated at a starting parasitemia of 0.2–0.5% in the presence of titration curves of the PmV inhibitors generated in this work. Incubation of parasites with synthetized compounds was in 96-well plates, testing triplicates of eleven points of serial ½ dilutions spanning 200 μM to 195 nM. The highest inhibitor concentrations gave a maximum DMSO concentration of ≤ 0.5%. Inhibition of growth after two full cycles (~ 3–4 days) was evaluated by flow cytometry. Cultures, live or fixed, were analyzed at day 3 and/or 4 after initiation, approximately~ 2 cell cycles of 3D7 strain. Parasitemia was measured by flow cytometry analysis of parasitized cells stained with 0.4 μg/ml Acridine Orange or 1 μM Topro 3 (Life Technology Invitrogen), using BD Fortessa or FACS Calibur instruments.

### Microscopy

Parasites were imaged live or fixed. Fixation in 4% formaldehyde and 0.0075% glutaraldehyde, cell permeabilization, antibody incubation, and mounting were as previously described [15, 23, 24, 30]. Before imaging of HRPII-GFP clone, we treated with **Compound 29** highly synchronized parasites at late trophozoite stage with inhibitors for 27 h, and then analyzed the distribution of the exported probe in newly invaded red blood cells. Images were acquired using a fluorescence microscope (BX51 Olympus). Live parasites were counter-stained using the nuclear dye Hoechst 33342 (Life Technologies—Molecular Probes).

### *In silico* analyses

#### Homology modelling of Plasmepsin V

Three on-line services for homology modelling were considered: Swiss Model [31], I-TASSER [32] and Phyre 2 [33] for the submission of the PmV amino acid sequence (UniProt ID Q8I6Z5). Models with a high score for confidence were compared in order to select the best model for molecular docking analysis. The volumes were evaluated by Fpocket [34] software integrated in the VEGA ZZ graphic environment [35]. The model obtained by Phyre 2 was selected because it had the largest volume catalytic pocket. This model yielded a confidence of 100%; it was derived from the structure of pro-plasmepsin of *Plasmodium vivax* (1MIQ) in PDB, which covers 35% of the Plasmepsin V sequence. The selected model was completed by adding the hydrogens, fixing the atom charges (Gasteiger—Marsili method) and the potentials (CHARMM 22), using the features included in VEGA ZZ software. The model was then optimized by NAMD 2 [36] (30,000 steps of conjugate gradients minimization) to reduce the high-energy steric interactions. This calculation was carried out by applying constraints to the protein backbone to avoid the collapse of the binding pocket. The secondary structure of the model was checked by calculating the Ramachandran plot, in which 54.24% were included in the most favored and 81.36% in allowed regions. Compatibility of the 3D model with its own amino acid sequence was evaluated by Verify3D [37]. The average score obtained for each amino acid was 0.18 (±0.28) with a positive score for 80.0% of the residues.

#### Modelling of the peptidic inhibitors

All peptides were built by the peptide builder tool included in the VEGA ZZ package [35], choosing β-sheet as the secondary structure, and considering the amino acidic side chains to be in ionic state, as would pertain in the biological environment. Resulting structures were optimized by NAMD 2.9 software [36] (5,000 steps, conjugate gradients method). The peptide structures were further optimized by semi-empirical calculations performed by PM7 method implemented in the MOPAC 2012 package [36]. All peptides were then collected in a database including 3D structures and several molecular descriptors calculated by VEGA ZZ and MOPAC 2012 (S1 Table); these were exploited together with the docking scores, to derive correlative equations to confirm the reliability of the ligand-enzyme complexes.

#### Molecular Docking

An induced fit procedure was initially performed using one of the smallest and most active peptides, *(S)* **-22**, employing the PLANTS software [38], keeping the enzyme rigid and considering the ligand as flexible. A 16 Å radius sphere was selected as the binding site, whose center is defined by Asp118 and Asp365, which are known to play a pivotal role in the catalytic process. PLANTS was set to use ChemPlp as the scoring function, to perform the pose search with the maximum exhaustiveness (speed1) and generate 20 clusters of structures (obtained by defining the RMSD value of 2.0 as clustering threshold). All complexes were graphically inspected and the best pose was selected by considering the docking score and the lowest distance between the amino and hydroxyl groups of Leu-HEA, in P_1_ position, from both catalytic aspartates. This complex was minimized by NAMD 2 [36], keeping fixed all the atoms outside a 12 Å radius sphere around the bound peptide. In order to favor the mutual adaptability between ligand and receptor, a molecular dynamics simulation was performed, consisting of an initial period of heating from 0 to 300 K (30,000 steps, 30 ps) and a 5 ns simulation phase with constant temperature, according to the Langevin’s algorithm. The Newton’s equation was integrated each femtosecond, according to Verlet’s algorithm and the frames were stored in the trajectory file every 5,000 iterations (5 ps). Particle Mesh Ewald (PME) for the electrostatic energy evaluation and periodic boundary conditions was also used, keeping the same atom constraints of the previous energy minimization. The first nanosecond of simulation was discarded because it was considered as the equilibration phase and in the remaining 4 ns the lowest energy structure was selected, this was optimized with the same parameters as the first minimization.

Each peptide was docked into the refined structure of PmV obtained by the induced fit procedure, setting the calculations with the same parameters of the preliminary docking study. The best complexes were minimized by NAMD 2 [36] (30,000 steps of conjugate gradients minimization) keeping fixed all residues not included in the spheroid defined by a layer of 10 Å thickness around the ligand. These refined models were then used to re-calculate PLANTS docking scores (ChemPlp, Plp and Plp95), APBS electrostatic binding energy, non-bond energies (CHARMM 22, CVFF and Coulomb), hydrophilic/hydrophobic interaction (MLP_InS_) and X-Score interaction scores (HPScore, HMScore, HSScore and pKd) using the VEGA ZZ package [35].

#### Computational Analysis

To evaluate the reliability of PmV structure obtained by homology modelling, the interaction scores of the complexes and the physicochemical properties of the peptides were considered, in order to find equations that predict the biological activity. The molecular properties and docking scores of inhibitors, which were tested biologically as a mixture of two epimers, were calculated as mean values of the contribution of each stereoisomer. Therefore, they were considered as a single molecular entity in the regression analysis. The automatic step-wise approach implemented in “Automatic linear regression” script of VEGA ZZ was used to build the regression models.

51 independent variables were considered and 15 of these were selected to build the models, due to their r^2^ ≥ 0.10. The multiple linear regression generates 1525 equations with a number of variables from 1 to 4. A preliminary leave-one-out cross-validation was carried out, allowing the resulting models to be evaluated in terms of r^2^ and predictive power (q^2^). Equations with three variables were preferred because the additional fourth descriptor does not introduce significant improvements in the statistic parameters. Equation 1 yielded the best statistics and it includes the variables: *Impropers* (number of pyramidal angles excluding those constrained by an aromatic ring), *Lipole* (lipophilic moment) and *MLP*_*InS3*_ (docking score that includes hydrophilic/hydrophobic complementarity between inhibitor and enzyme). The prediction strength of the best equation was evaluated by both leave-one-out cross validation, and by splitting randomly the whole dataset into 20 pairs of training and test sets, including 40 and 20 molecules, respectively. For each training set, the regression coefficients were calculated to evaluate the test set, in terms of standard deviation of errors, angular coefficient, intercept and r^2^ of the trend line of the chart of the predicted vs. experimental activities. This validation was performed automatically by the “Model validator” script included in VEGA ZZ package [35]. For each training set, new regression coefficients were calculated to evaluate the test set. For each pair, the training set was used to predict the activity of the test set, obtaining mean r^2^ of the trend line of the predicted vs. experimental activity plot of 0.72 (±0.07), comparable to that of Equation 1 (0.74). Moreover, for all independent 20 training sets, the average mean r^2^ value was 0.75 (±0.03).

## Results

### Transition state inhibition of Plasmepsin V

**Fig 1:**
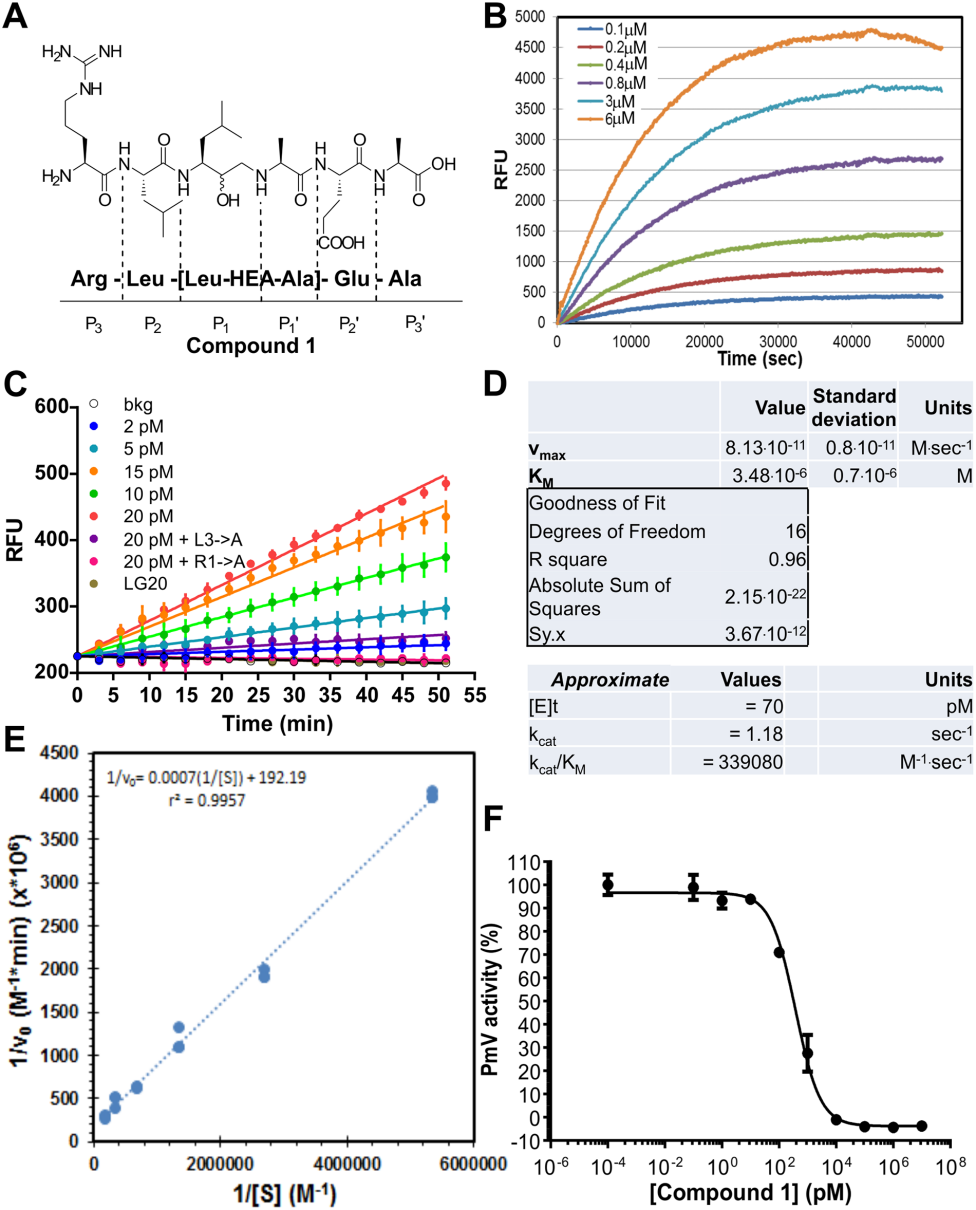
Plasmepsin V activity and inhibition. **(A) Compound 1**: chemical structure, amino acid composition and correspondence to the PExEl substrate P and P' positions. The PExEl motif is known as RxLx(x) ^E,Q,D^ [7] and is processed by PmV, downstream to the third leucine [14, 15, 40]. [Leu-HEA-Ala] indicates the presence of the group ((3S)-3-amino-2-hydroxy-5-methylhexyl)-L-alanine. This fully resembles the leucine and alanine in the third and fourth positions of the PExEl motif, except for the hydroxyethylamino group linking the two simil-amino acids in place of a peptidic bond. **(B-E)** Plasmepsin V activity and functional validation. (B) Cleavage of *DABCYL*-LNKRLLHETQ-*EDANS*, HRPII-derived fluorogenic substrate, by PmV at different concentrations of substrate (from 0.19 to 6 μM), to completion (means of triplicates are shown). Micheal-Menten analysis of initial velocities resulted in *K*_*M*_ of 3.48 (±0.7) 10^−6^ M for *DABCYL*-LNKRLLHETQ-*EDANS*. (C) Activity (RFU, relative fluorescent units) was measured in a 96-well plate format for ~50 min after addition of fluorogenic substrate (final concentration ~3 μM). Lines represent linear regressions of data obtained in triplicates. Standard error bars are shown at 3 minute intervals. The black line corresponds to background fluorescence; dark blue, light blue, green, orange and red lines are the activity of PmV’s titration in the order from lowest to highest concentration, approximatively 2, 5, 10, 15, 20 pM; purple and pink lines represent the activity of 20 pM PmV against fluorogenic peptides containing critical mutations of the PExEl motif: L3 → A and R1 → A, respectively; the golden line shows inhibition of 20 pM PmV in the presence of our inhibitor **1** at ~2 μM. (D) Enzymatic parameters (K_M_ and V_max_) were calculated via non-linear curve-fitting analysis taking into account the substrate concentrations and the initial velocities (v_0_). These were derived from activity curves as shown in (B), acquired in triplicates (as in Materials and Methods). Active enzyme concentration [E]_t_ and, consequently, k_cat_ (calculated) are only approximate values possibly affected by significant errors due to the limits of the applied quantization methodology for active PmV (densitometry of protein gels). Variations of these values in the range of 2–3 fold have been detected in diverse kinetic measurements. (E) A Lineweaver—Burke plot is shown derived from triplicates of PmV-cleavage HRPII-derived fluorogenic substrate, over time at different concentrations of substrate (from 0.19 to 6 μM). (F) PmV activity inhibited by **Compound 1**. On semi-logarithmic plot a typical titration curve obtained with serial dilutions of **1** is shown. Inhibitor IC_50_ was calculated by analysis of the sigmoidal fitting of the data. Error bars represent SEM of data.

At the start of this work the available tridimensional structures of aspartic proteases were used to generate 3D-models of *P*. *falciparum* Plasmepsin V (gene code PF3D7_1323500, former identification, PF13_0133) via homology modelling platforms [31–33]. Models were constructed using both the full length *P*. *falciparum* Plasmepsin V (Pf_PmV) and just its catalytic domain (amino acids 81–500), giving similar results. Two elements were consistently found in all the models: a structural unpredictability of peculiar PmV subdomains that have no or very low homology to other known proteases [17], and a folding of the catalytic domain into two independent subunits. This type of folding is a common trait of aspartic proteases and forms the active site by juxta-positioning the two active aspartates (in Pf_PmV: Asp118, 365), that are contained in each of the subdomains. Despite the high confidence score of the predictions, PmV 3D-models proved to be highly heterogeneous, showing great diversity for the predicted catalytic grooves and no rational criteria for selecting a reference model were suitable (data not shown). In fact, due to the peculiarity of Pf_PmV, none of the analyzed proteases presented a significant similarity to Pf_PmV, yielding values for identity in the range of 18–29%, and homology from 7e^-4^ to 8e^-8^ [33]. *Plasmodium* PmV uniqueness was confirmed by the structural data of *P*. *vivax* PmV, a closest homolog of Pf_PmV, that was recently published while this paper was under revision [39]. As the structural uncertainty of the catalytic domain predictions was significant, in order to generate inhibitors we opted for designing inhibitors incorporating minimal modifications of the PmV natural substrate, the PExEl motif (Fig 1a). These molecules were then used to chemically scan PmV catalytic site accessibility and requirements for inhibition.

PmV was purified directly from parasites [15]. Each enzyme batch was routinely assayed for purity, specificity and steady-state enzymatic parameters using fluorogenic peptides containing the wild-type or the mutated PExEl-motif [15] (examples in Fig 1b–1e). We consistently calculated a K_M_ of 3.48 (±0.7) μM for the HrpII-PExEl substrate. In order to generate PmV-inhibitors, we synthesized molecules that resemble the PExEl sequence, RxLx(x)^E,D,Q^ [7] and contain the proteolytically uncleavable hydroxyethyl-amino group (HEA) [41] that was positioned downstream of the natural PmV cleavage site [7, 15, 40]. Our synthetic molecule LG20 (from now on referred in this work as **Compound 1**) (Fig 1a) is composed of six PExEl-like amino acids, RL[L~A]EA, where ‘L~A’ resembles the third and fourth positions of the PExEl motif. ‘L~A’, ((3S)-3-amino-2-hydroxy-5-methylhexyl)-L-alanine, mimics the aspartic protease transition state [41]. Using our previously published *in-vitro* assay for Plasmepsin V [15], we showed that **Compound 1** efficiently inhibits PmV with an IC_50_ (half maximal inhibitory concentration) of 367 (±60) pM (Fig 1f). This value was independent of variations in the preparation of the inhibition assay. Pre-mixing the enzyme with inhibitors or adding the inhibitor after the reaction was initiated, gave IC_50_ values which were substantially unaltered, respectively, 367 and 328.9 pM. The K_I_ of **Compound 1**, calculated from the values of the IC_50_ and the enzyme K_M_, was estimated at 0.197 (±0.07) nM, currently the highest reported affinity for PmV.

**Fig 2:**
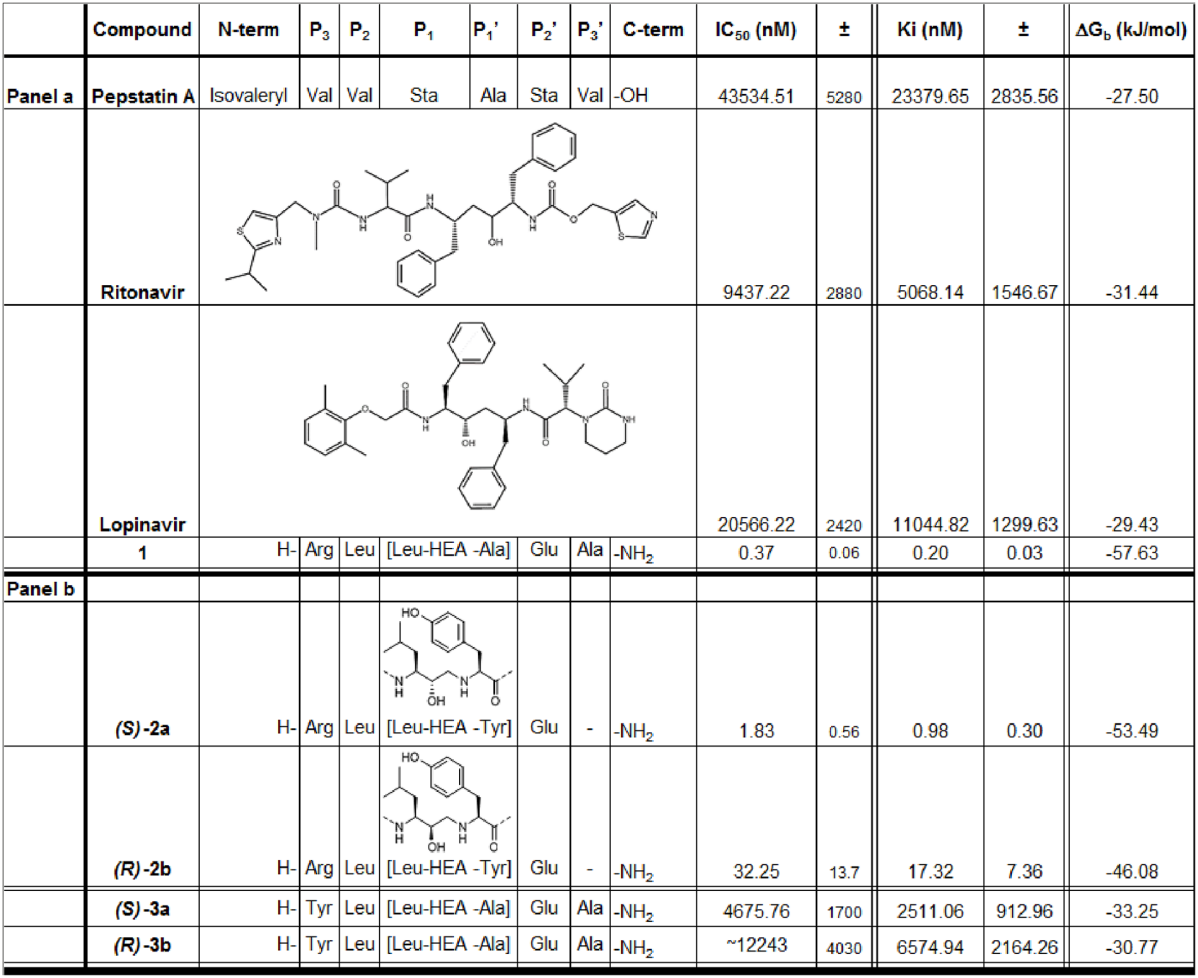
PmV inhibitory activity of the new compounds. IC_50_ values, obtained in the presence of 3 μM of HRPII-PExEl substrate, are shown. They are averages of not less than three independent inhibition curves. Standard errors of the calculation of the IC_50_s by sigmoidal fitting are shown in column ‘±’. HEA indicates the hydroxyethylamino group that links P_1_, the non-amino acidic leucine analog, (3S)-3-amino-2-hydroxy-5-methylhexyl- group, and P_1_', the alanine. Sta is statine, an atypical amino acid found in the natural product Pepstatin A. **Panel a** Activity comparison of the newly generated inhibitor **1** with previously tested compounds [14, 15]. **Panel b** Activity of two generated diastereoisomers presented in Fig 4. Chemical structures of HIV inhibitors and absolute chirality of Leu-HEA epimers is included. Chemical structures of Lopinavir and Ritonavir are included in panel a; and stereoisomers of Leu-HEA-Ala are shown in panel b.

**Fig 3:**
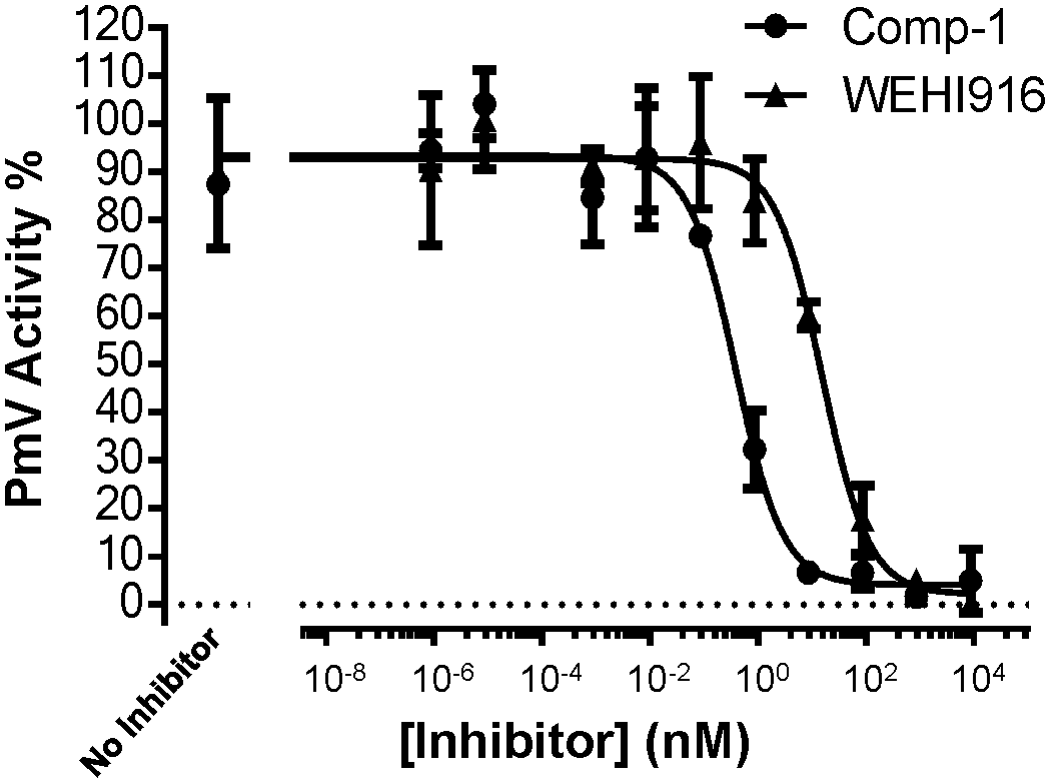
Comparison between different inhibitors of PmV activity. Curves represent the sigmoidal fittings of the data, obtained in triplicates, on PmV inhibition by either **Compound 1** (●) or WEHI916 (▲). Data were fluorescent readings from a single experiment, where both inhibitors were simultaneously analysed. Standard error bars (SEM) are included. In this experiment the calculated relative IC_50_s were 367 pM for **Compound 1** and 32.43 nM for WEHI916.

Inhibitors of Pepstatin A and HIV-protease have been previously reported as inhibitors of PmV activity [14, 15]. We, therefore, assayed Pepstatin A, Lopinavir and Ritonavir in parallel to **Compound 1** obtaining for these molecules IC_50_s in the range of 9–40 μM (Fig 2 panel a). WEHI916 is another inhibitor of Plasmepsin V, published by Prof A. Cowman’s group [19, 20] while our work was in preparation. Therefore, we also compared side-by-side, **Compound 1** and WEHI916, in order to test the IC_50_-relative differences in the same assay conditions. In our assay format, WEHI916 yielded IC_50_ of 32.43 (±1.43) nM, close to the published value of 19–20 nM [19, 20], against a confirmed picomolar inhibitory efficiency of **Compound 1** (Fig 3). As discussed below, both **Compound 1** and WEHI916 are transition-state inhibitors, but with different scaffolds, the first being based on a HEA group, the second on a statine.

**Fig 4:**
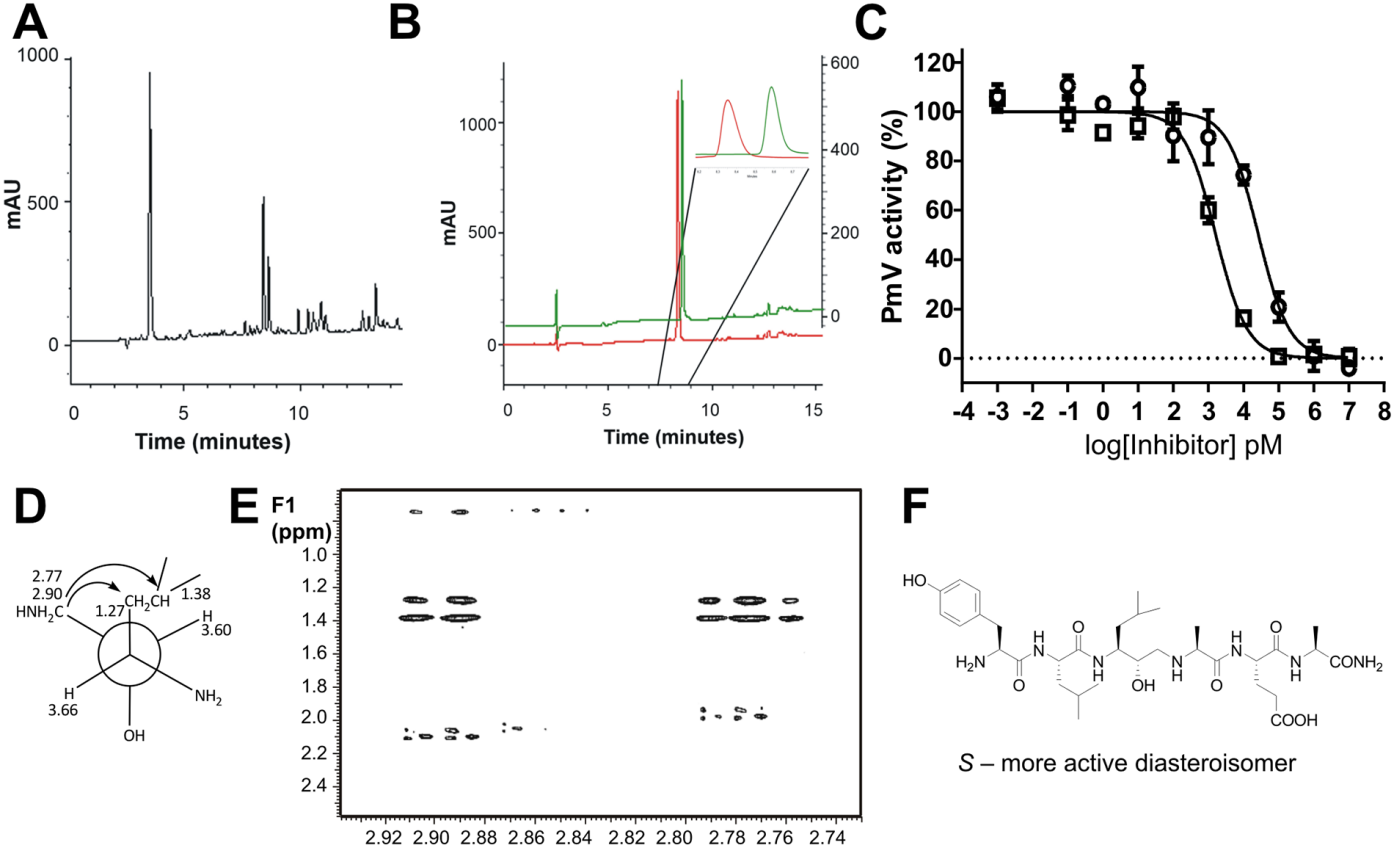
Separation and activity of the synthetized diastereoisomers. **(A)** Analytical RP-HPLC chromatogram of **Compound 2** crude reaction mixture. **(B)** Superimposition of RP-HPLC traces of **Compound 2a** (red) and **Compound 2b** (green) after preparative purification. Inset: enlargement of the two separated diastereoisomers. **(C)** Inhibitory activity of PmV activity by **Compound**s **2a** and **2b** (Fig 2 panel b). Bars represent SEM. **(D-F)** NMR analysis. (D) Key cross-peaks for the 2D NMR ROESY spectrum. (E) The relevant section of 2D NMR ROESY spectrum of **Compound 3a**. (F) Absolute stereo-structure of the active diastereoisomer. Configuration at the carbons was determined through *J*-based configuration analysis. Selected cross-peaks detected in the 2D NMR ROESY spectrum supported the stereo-chemical assignment.

We then evaluated the accessibility of PmV active site using a panel of potential inhibitory molecules, generated by modifying **Compound 1**. In order to assess the relative inhibitory activity of the generated molecules, the previously published *in-vitro* assay for Plasmepsin V [15] was adapted for high density multi-well plates. A fast and automatable synthesis protocol for the molecular scaffold of **Compound 1** was created; this easily allowed the introduction of multiple variations to the P and P' regions (Fig 1). A panel of ~70 molecules carrying modifications of N- and C-termini, amino acid R1 groups and size with respect to **Compound 1**, was successfully generated. Synthesized molecules were purified up to 95.1–99.9%, physico-chemically assayed and tested for PmV inhibitory activity as detailed in the Materials and methods (Fig 4a and 4b).

Our synthetic protocol results in mixtures of two diastereoisomers of the final compound and we detected that maximum PmV inhibition was due to one of the two generated diastereoisomers. These differ in the configuration of the new stereogenic carbinol center of the HEA group (Fig 1a). When technically feasible, the two diastereoisomers (Fig 4b) were isolated by RP-HPLC and independently tested for PmV inhibition. The first RP-HPLC-eluted stereoisomer, referred as peak “a”, was 10–100 times more active than the second peak (“b”). Activities of **Compound 2**’s diastereoisomers are shown as an example in Fig 4c (Fig 2 panel b). Through 2D-NMR ROESY spectroscopy (via *J*-based configuration analysis [22]), the configuration of the HEA moiety was assigned. Since the leucine configuration was known, the analysis of selected cross-peaks revealed that the absolute configuration of HEA’s carbinol group was unambiguously *S* for the more active diastereoisomer (as example, **compound 3a** is shown in Fig 4d–4f).

### SAR analysis on the enzymatic PmV inhibition

All compounds, and their isolated diastereoisomers, were tested *in vitro* for inhibition of PmV activity. If no separation of the two diastereoisomers was possible, the tested activity was assumed to be due to a mixture of the two. The results of our SAR analysis on the enzymatic PmV inhibition are presented relative to each of the modifications introduced into **Compound 1**.

#### C-Terminus modifications

**Fig 5:**
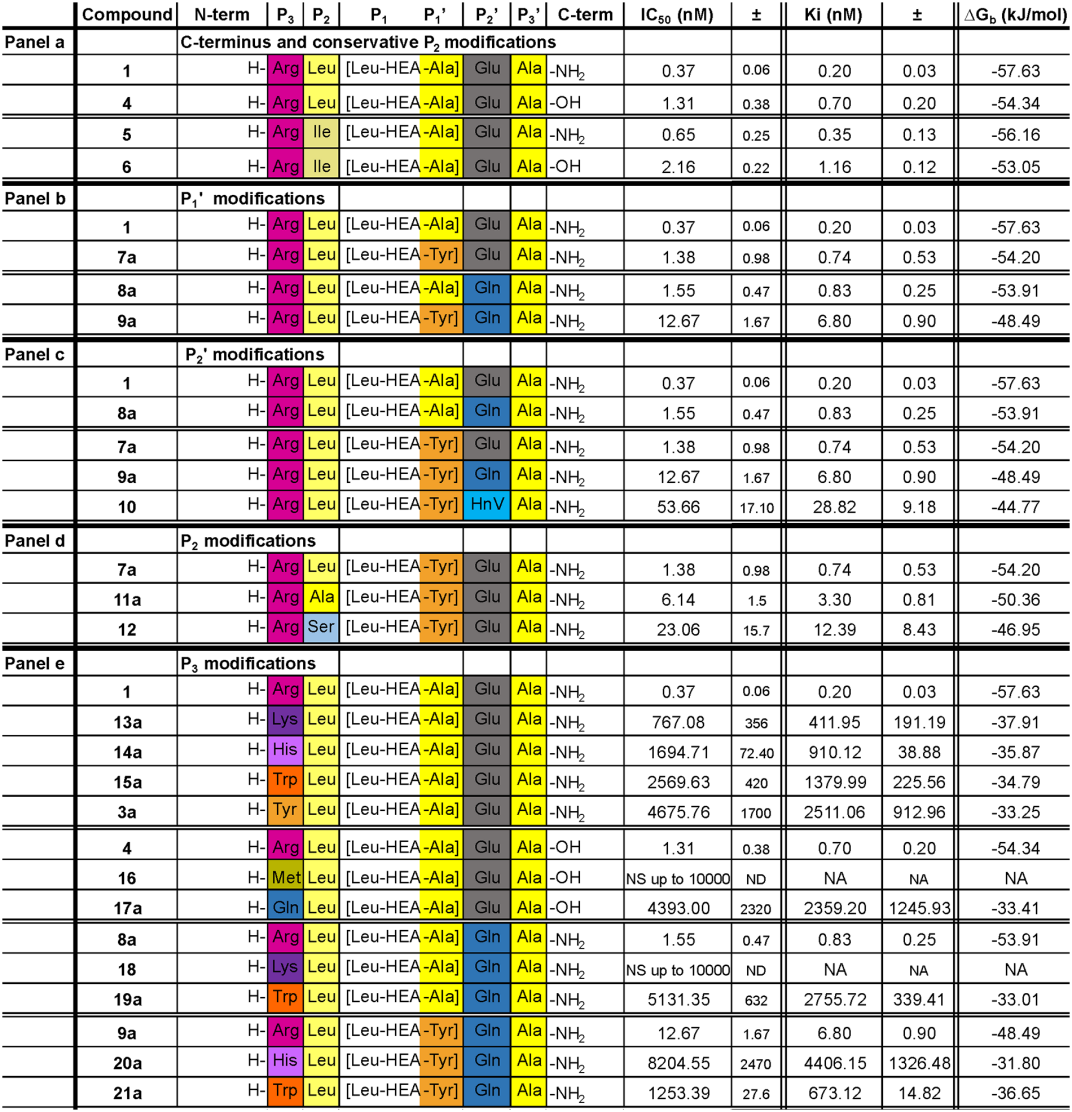
PmV inhibitory activity of the generated compounds (1^st^ set). IC_50_ values, obtained in the presence of 3 μM of HRPII-PExEl substrate, are shown. They are averages of not less than three independent inhibition curves. Standard errors of the calculation of the IC_50_s by sigmoidal fitting are shown in column ‘±’. HEA indicates the hydroxyethylamino group that links P_1_, the non-amino acidic leucine analog, (3S)-3-amino-2-hydroxy-5-methylhexyl- group, and P_1_', the alanine. HnV is hydroxynorvaline, a non-natural amino acid. Relevant modifications of **1** as follows: **Panel a**, C-terminus; **b**, P_1_'; **c**, P_2_'; **d**, P_2_; **e**, P_3_. Amino acids are three-letter and colour coded except for ‘[Leu-HEA-…]’ corresponding to the (3S)-3-amino-2-hydroxy-5-methylhexyl- group, as follows: hydrophobic amino acids (aa) are in shades of yellow; aromatic aa, orange; positively charged aa, purple; polar aa, blue; and acidic aa, grey. NS = Not significant; ND = not determined; NA = not applicable.

By amidating the C-termini (mimicking the elongation of the P' peptidic backbone), IC_50_s increase slightly (1.3–3.5 times), as is consistently seen in **Compounds 4** and **6** in comparison to **1** and **5**, respectively (Fig 5 panel a). A similar trend was later recorded for other inhibitors in which this modification was tested, as described below.

#### P_1_' modifications

P_1_' is one of the less well conserved positions of the PExEl motif. In this position, we tested the introduction of an aromatic amino acid as tool for improving peptide purification, and quantification procedures relying on tyrosine absorbance. This substitution yielded a limited increase (4–7 fold) of IC_50_s, possibly due to augmented steric hindrance, as seen in **Compound 7a** with respect to **Compound 1**, and **9a** compared to **8a** (Fig 5 panel b).

#### P_2_' modifications

Exchanging glutamate to glutamine in P_2_', a position fairly well conserved in the PExEl motif [7], the loss of the negative charge reproducibly caused a slight reduction of activity. We detected a minimal perturbation of the inhibitory activity, giving an IC_50_ increase of ~4 times in **Compound 8a** compared to **Compound 1** (Fig 5 panel c). In the presence of tyrosine in P_1_' (**9a** compared to **7a**), the effect on the inhibitory activity for the same modification was twice as pronounced (~9 fold). 5-Hydroxynorvaline (HnV) was then introduced in P_2_', this is a non-natural amino-acid corresponding to reduced glutamate (**Compound 10)**. Despite missing both the carboxyl moiety and the negative charge of the Glutamate, this molecule still inhibits the enzyme in the nanomolar range, showing an IC_50_ increase of no more than 5 fold compared to **9a** (Fig 5 panel c). Other modifications of this site were tested, as described below.

#### P_2_ modifications

A minimal increase of the IC_50_ was detected when P_2_ leucine was replaced with isoleucine, as seen in **5** compared to **1** and **6** to **4**. This is possibly due to the presence of a catalytic S_2_ pocket slightly more permissive to methyl branches on the distal carbon than on C2 (Fig 5 panel a).

Using a set of inhibitors containing tyrosine in P_1_', we assessed the importance of the hydrophobic interactions of the leucine in P_2_. By either minimizing the hydrophobic chain, using alanine (**11a**), or introducing a hydroxyl (**12**), we observed an increase in IC_50_ levels of 5 and 15 times, respectively (Fig 5 panel).

#### P_3_ modifications

P_3_ arginine is an essential component of PExEl substrates and its substitution to lysine affects both protein processing and export [14, 15] (Fig 1c). Nevertheless, we decided to screen the degrees of freedom in the S_3_ binding pocket by testing various substitutions in order to explore alternatives to the high hydrophilic profile of **Compound 1**. All our P_3_ modifications produced deleterious effects on inhibition of PmV, independent of the type of polar tail used to substitute the arginine side chain. However, surprisingly many of these modifications still resulted in inhibitory molecules with a potency in the low micromolar range, performing significantly better than HIV inhibitors (Fig 2 panel a). In particular, while IC_50_ increases up to 0.77 μM with lysine (**13a**), the presence of histidine (**14a**), tryptophan (**15a**), tyrosine (**3a**), glutamine (**17a**) or methionine (**16**) in P_3_ decreased the inhibitory activity progressively (Lys>His>Trp>Tyr>Gln) up to the completely inactive **Compound 16** (Fig 5 panel).

#### Shorter scaffolds (ΔP_3_')

**Fig 6:**
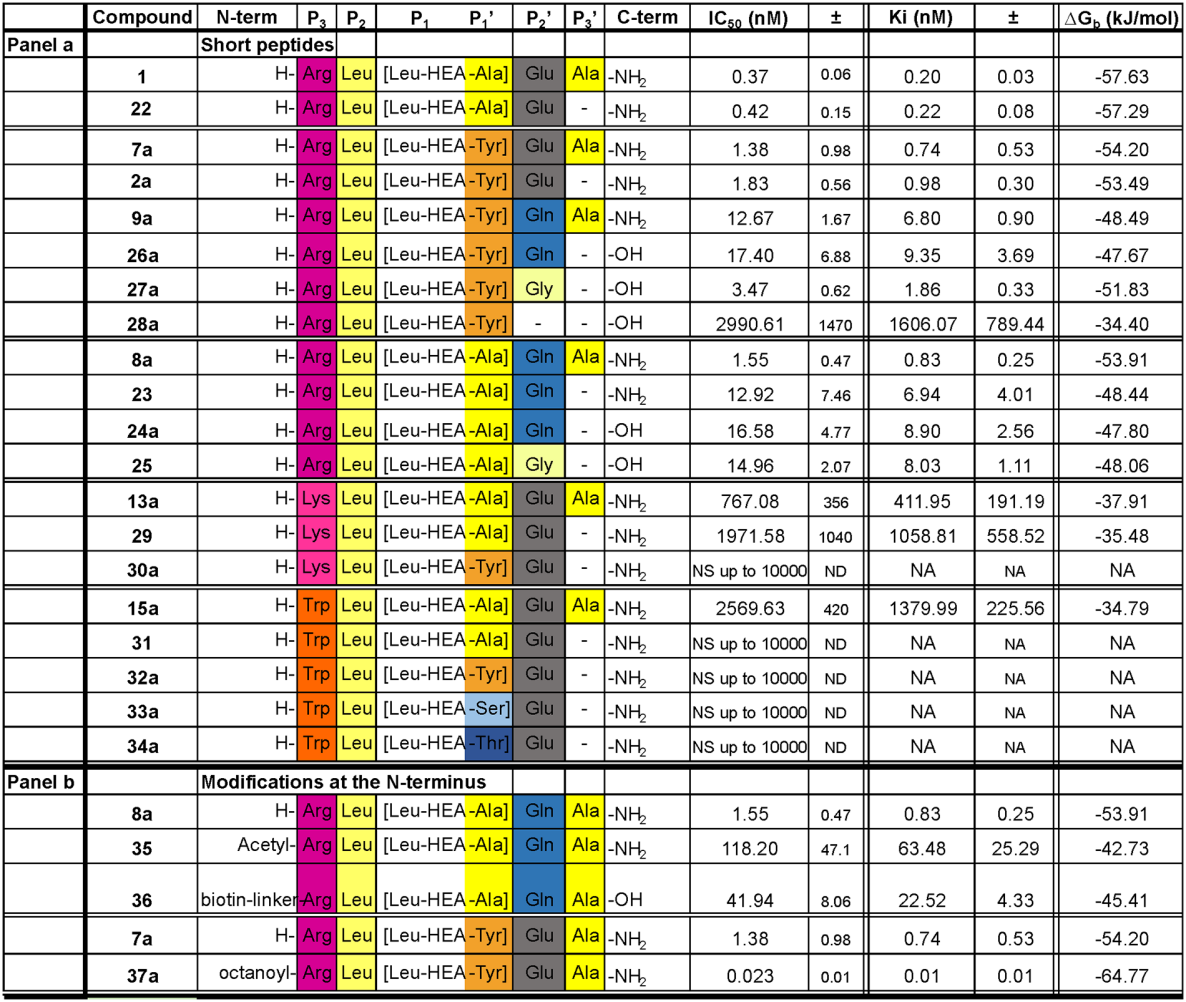
PmV inhibitory activity of the generated compounds (2^nd^ set). IC_50_ values, obtained in the presence of 3 μM of HRPII-PExEl substrate, are shown. They are averages of not less than three independent inhibition curves. The standard errors of the calculation of the IC_50_s (by sigmoidal fitting) are shown in column ‘±’. HEA indicates the hydroxyethylamino group that links P_1_, the non-amino acidic leucine analog, (3S)-3-amino-2-hydroxy-5-methylhexyl- group, and P_1_', the alanine. Relevant modifications follow: **Panel a**, Shorter molecules; **b**, modifications of the N-terminus. The dash at a given position indicates absence of the amino acidic residue in that position. Amino acids are three-letter and colour coded but ‘[Leu-HEA-…]’ corresponding to the (3S)-3-amino-2-hydroxy-5-methylhexyl- group, as follows: hydrophobic aa are in shades of yellow; aromatic aa, orange; positively charged aa, purple; polar aa, blue; and acidic aa, grey. Two GABA units were used as linker for the Biotinylated **Compound 36**. NS = Not significant; ND = not determined; NA = not applicable.

Bioinformatics analysis of the PExEl motifs in *P*. *falciparum* shows high variability for the P' region [7]. A region expected to be less determinant for enzyme affinity. Therefore, we decided to progressively trim this region in order to minimize the dimensions of our molecules and to test the importance of this region for inhibition. We first studied the importance of the alanine in P_3_' within compounds containing either alanine or tyrosine in P_1_'. In both cases, when P_3_' alanine was removed approximately the same activity was detected, as shown by **Compound 22** compared to **1**, **2a** to **7a** and **26a to 9a** (Fig 6 panela). More pronounced increases in IC_50_ (2.5 and 8 fold) were observed in **Compounds 23** and **29** when compared to their closest cognate molecules **8a** and **13a**. However, we consider these differences to be of lesser significance, as the comparison is between pure ‘a’ diastereoisomers and racemic mixtures. The amidation of the new C-terminus does not substantially change the activity (**24** compared to **23**).

All the shorter peptides carrying tryptophan in P_3_ (**Compounds 31**, **32a**, **33a** and **34a**), independently of the nature of the residue in P_1_', lose inhibitory activity up to 10 μM. This suggests that the residual inhibitory activity of the molecules lacking the arginine in P_3_ strongly depends on the overall P' interactions. Interestingly, the inhibitory activity was still retained in shorter inhibitors with the lysine in P_3_ (**Compound 29**). However, both lysine in P_3_ and tyrosine in P_1_' (**30a**) lose inhibitory activity up to 10 μM (Fig 6 panela).

#### Shorter scaffolds (ΔP_3_'-P_2_’)

We then tested the removal of the P_2_'. When we deprived P_2_' position of its side chain by introducing a glycine, the smallest of the natural amino acids, the activity surprisingly remained almost unaltered, as shown by comparison of **Compounds 25** to **24**, and **27a** to **26a** (Fig 6 panela); this was suggestive of the dispensability of the P_2_' position. However, when we tested **Compound 28a**, lacking both amino acids, the inhibitory activity was severely decreased ~ 1,000 times, indicating the loss of essential interactions possibly due to the glycine carbonyl group (Fig 6 panela).

#### N-Terminus modifications

We synthetized modifications at the N-terminus to either increase the lipophilic properties of the molecules, or to perform biochemical analyses. Acetylation of the N-terminus produced some loss in PmV inhibition activity (**35** compared to **8a**), similar to what was observed when the biotin derivative **Compound 36** was tested (Fig 6 panelb). In both cases, pure diastereoisomers were not isolatable and their stereogenic purity could not be estimated. Loss of activity was not significant, remaining in the range of the low nanomolar. **Compound 36** was later used for biochemical and cellular experiments, as described below, since it contains a biotin group.

Surprisingly, the introduction of the octanoyl group (**37a**) at the N-terminal resulted in an extremely potent inhibitor with IC_50_ of ~ 23 pM, more than 50 fold more active than its parent compound **7a** (Fig 5 paneld and Fig 6 panel b). This suggests the presence of a lipophilic environment in areas surrounding PmV S_3_, that could be the cause of the potency increase of the inhibitor by accommodating the hydrocarbon tail.

### *In-silico* analyses

#### Molecular docking

**Fig 7:**
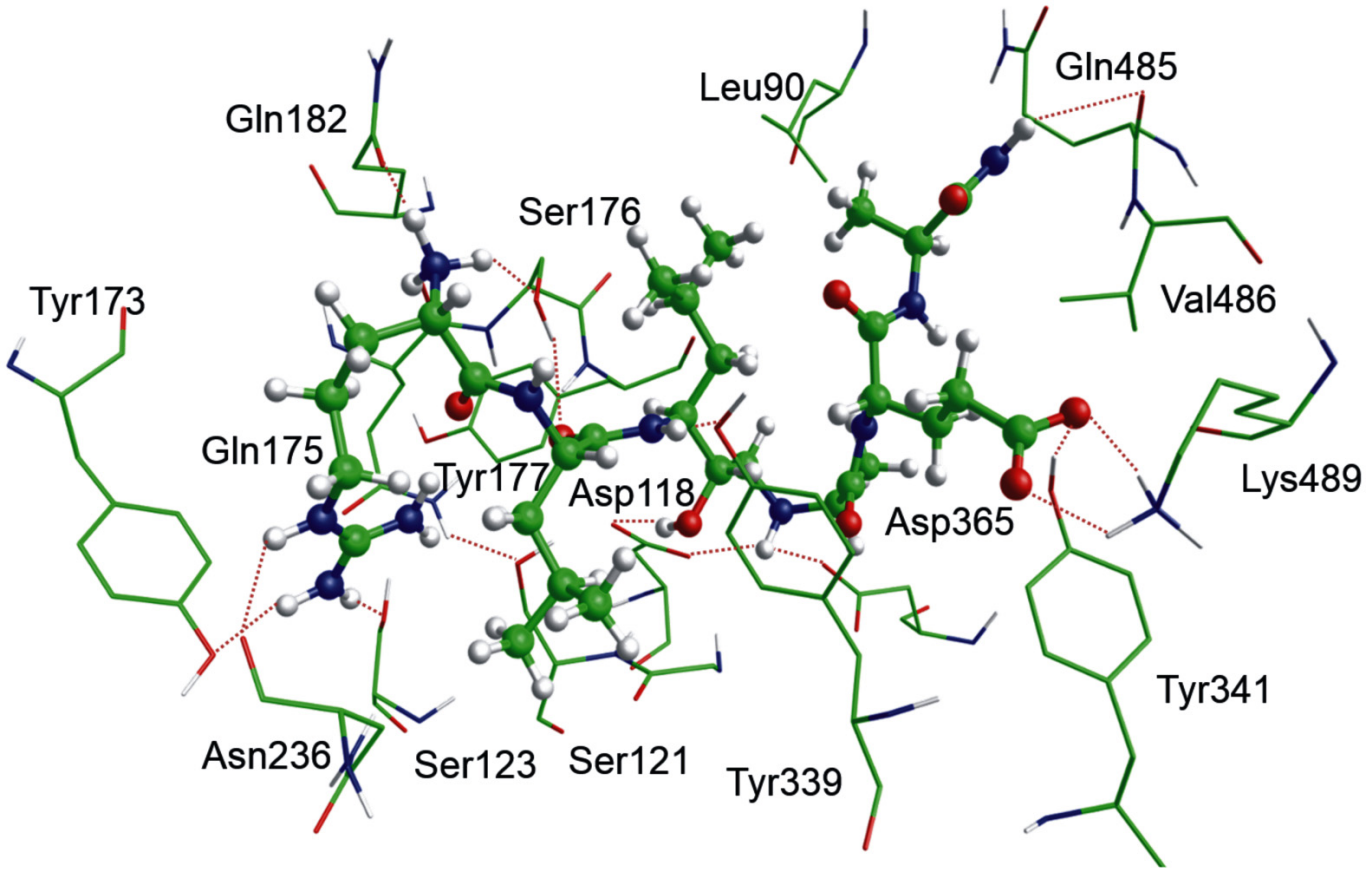
In-silico 3D model of PmV active site bound to *(S)*-1. 3D representation of *(S)*-1-PmV complex was obtained by Phyre 2 homology modelling software. **1** is represented as solid ‘balls and sticks’ while PmV residues surrounding the catalytic pocket are as wireframe (C, N, O, H are respectively green, blue, red and white). Only hydrophilic hydrogens are included. For sake of clarity, only the best interacting residues are shown and the dotted lines indicate the presence of significant intermolecular interactions. 3D animation model is in S2 File; and 2D scheme of the main interactions is in Fig 8.

**Fig 8:**
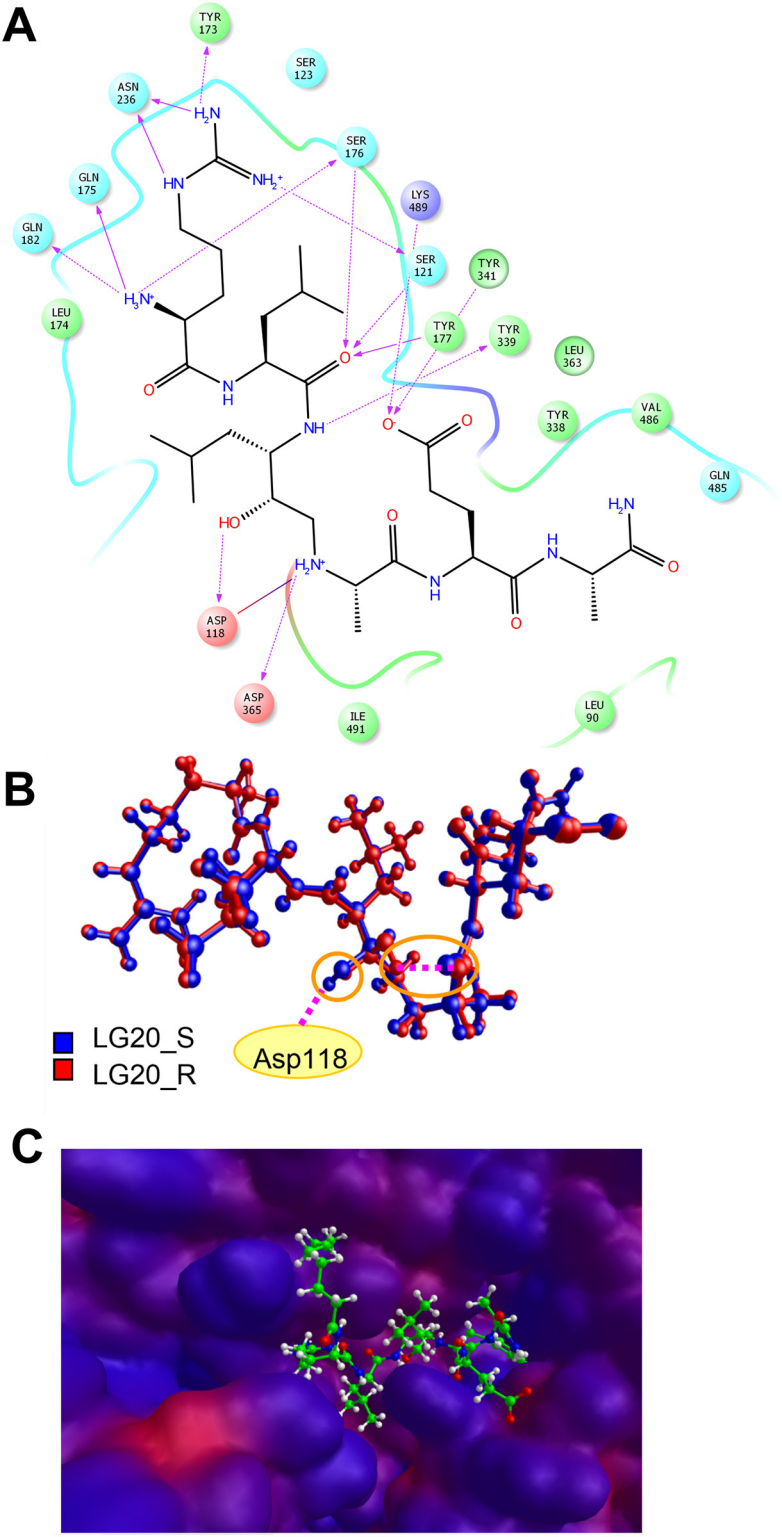
Modelling data. **(A)** 2D scheme of the main interactions between *(S)-* **1** and Plasmepsin V obtained by Maestro 9.7 software (Schrödinger, LLC, New York, NY, USA). Solid arrows indicate H-bonds with the backbone; dashed arrows, H-bonds with the side chains; and purple lines, ionic interactions. **(B)** Superimposition of both **1** epimers kept in their original docking pose. The R stereoisomer (red) is stabilized in the turn-like conformation via an intramolecular H-bond, but loses the H-bond interaction with Asp118 of *S* stereoisomer (blue). No other differences were found in the binding mode of both epimers. **(C)** Lipophilicity surface of Plasmepsin V complexed with *(S)*-**37** peptide. This is calculated by the Molecular Lipophilicity Potential (MLP) [42] implemented in VEGA ZZ software [35]. The hydrophilic and hydrophobic regions are shown, respectively in red and blue. In close proximity to S_3_ large hydrophobic areas are noticeable, one of which accommodates the octanoyl moiety of **Compound 37a**.

We performed *in-silico* studies of the binding between our inhibitors and available PmV 3D models in order to analyze their binding to PmV and allow chemical scanning of enzyme binding pockets. For this purpose several PmV 3D models were screened for prediction confidence, consistency and accessibility of the catalytic space. The model obtained by Phyre 2, which was derived from the structure of pro-plasmepsin of *P*. *vivax* (1MIQ in PDB), was selected as the starting point for the docking of inhibitor *(S)*-**1**. Molecular dynamics, performed to increase the fit between the interacting partners, yielded inhibitor *(S)*-**1** as being stabilized by an extensive H-bonds network. This network was common to all the complexes obtained with the other compounds (Figs 7 and 8a, S1 and S2 Files). Leu-HEA plays a pivotal role in binding by interacting with both carboxyl groups of the active aspartates via its secondary amine. The inversion of the chiral center (from *S* to *R*) causes the loss of a key H-bond with Asp118 (Fig 8b) but no other significant changes in binding for the rest of the molecule. Therefore, this chiral inversion seems to be tolerated, correlating with the fact that some R epimers yielded reduced but still significant inhibitory activities in the low nanomolar range (for example IC_50_ of 1.57 nM detected for **37b**, 188 nM for **8b**, ans **2b** (in Fig 2 panel b)).

**Fig 9:**
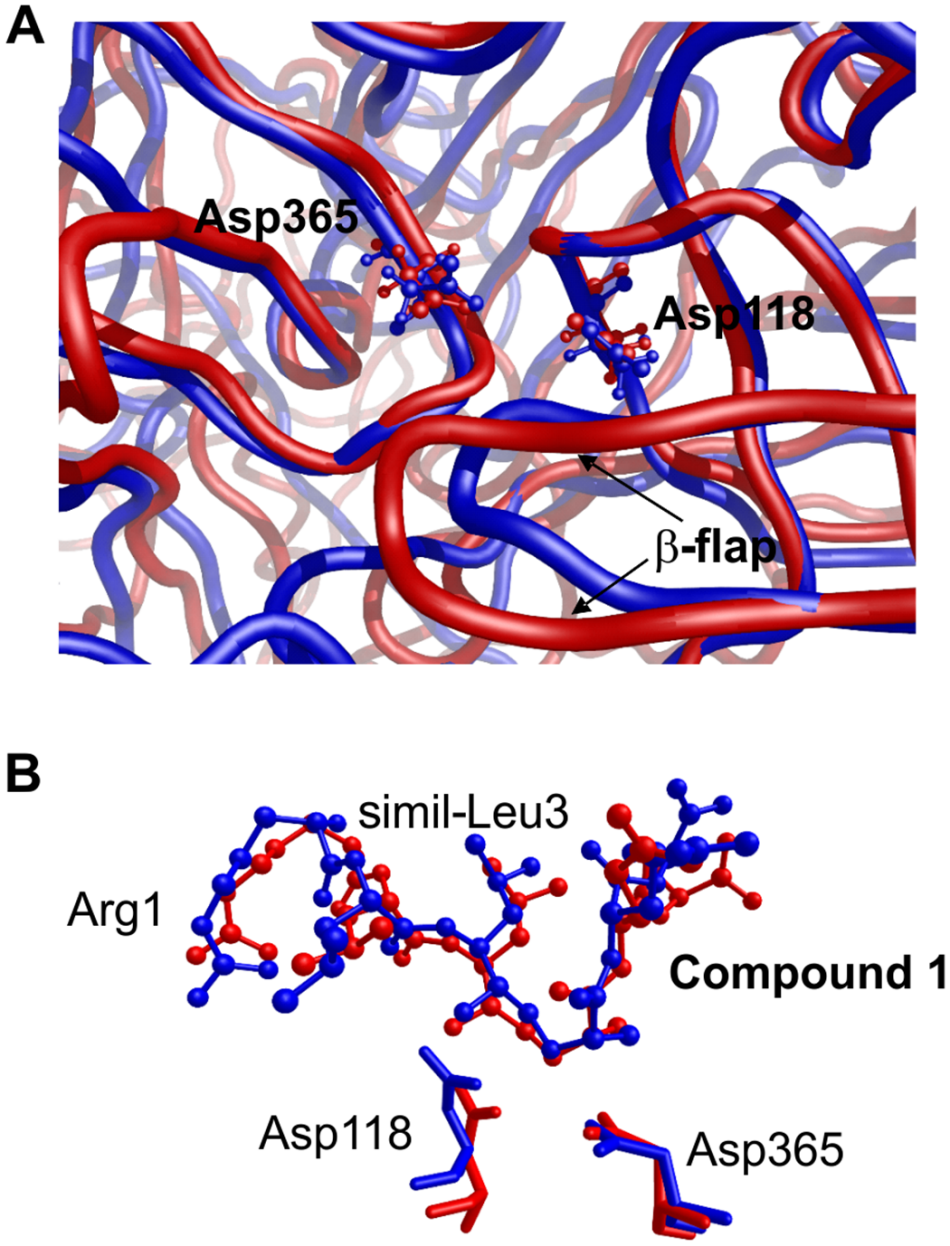
*In-silico* comparison of the 3D models of PmV. **(A)** Superimposed catalytic domains of two Pf_PmV 3D models. The one we used in this work (blue ribbon) (PDB file in S1 File) and the one derived from modelling Pf_PmV to the recently published structure of Pv_PmV in complex with WEHI-842 (red ribbon) [39] (PDB file in S3 File). 3D models of PmV were obtained by Phyre 2 homology modelling software using as templates either pro-plasmepsin of *Plasmodium vivax* (PDB code 1MIQ) (blue ribbon) or the recently published Pv_PmV in complex with WEHI-842 (PDB code 4ZL4) (red ribbon) [39]. The two 3D models were then superimposed, aligning the alpha Carbons of the peptidic backbones. The two catalytic aspartates and the β-sheet flap, that form the aspartic protease catalytic groove, are indicated. **(B)** *In-silico* comparison between *(S)*-**1** bound to each of the two 3D models. 3D representation of *(S)*-**1**-PmV complex was obtained by Phyre 2 homology modelling software. The *(S)*-**1**-PmV complex to the model used in this work is in blue, while the complex to the model derived from Pv_PmV is in red. **Compound 1** is represented as solid ‘balls and sticks’ while PmV active aspartates of the catalytic pocket are as wireframe. For sake of clarity, only **Compound 1** (indicating the principal residues, R1 and L3), Asp118 and Asp365 are shown. The superimposition of the full 3D models is available in S3 Fig.

The residue in P_3_ position can elicit several H-bonds, inserting in an electronegative pocket where the proximity of aromatic amino acids, such as Tyr173, has been noted. Therefore, P_3_ can interact via either charge-transfer or π-π stacks with positively charged and aromatic residues, if present. Additional ionic interactions can take place with residues of the β-hairpin flap (Glu179 and Glu215), which is expected to hang over the active site. The P_3_ N-terminal nitrogen can elicit interactions with a small accessory hydrophilic pocket. These interactions are partially lost when acetyl or octanoyl moieties are introduced. However, in close proximity to the S_3_ we detect lipophilic areas, one of which is occupied by the side chain of the amino acid in the P_2_ position. The other areas can elicit interactions with lipophilic groups, such as the octanoyl group (Fig 8c). The side chain of the tyrosine in P_1_' can be placed in the catalytic site, without showing significant changes due to steric hindrances. The P_2_' side chain, if charged, can interact ionically with Lys489 and, via an H-bond, with Tyr341. P_3_' does not appear to play an important role in interaction. Recently, while this paper was under review, the tridimensional structure of *P*. *vivax* Plasmepsin V (Pv_PmV) was published [39], revealing novel structural features for this unique aspartic protease that are possibly required for its particular cellular activity [43]. *P*. *falciparum* and *vivax* PmV, showing identity of 52.6% and similarity of 68.1% (S3 File) are sufficiently different to significantly hinder Pf_PmV expression and crystallization, indicating that there are some critical differences between the two enzymes. However, when we superimpose the alpha carbons of the residues included in the catalytic domain of the model we generated in this work (S1 File) to the tridimensional structure, generated via homology modelling using the recently published Pv_PmV structure as template (4ZL4 in PDB) (S4 File), we note that the two domains appear very similar (Fig 9a) with a root mean square deviation of 0.948 Å. We also performed a docking analysis of **Compound 1** to the model derived from Pv_PmV, confirming that little modification of the poses of **Compound 1** was observed (Fig 9b). In this case, a root square mean deviation of 1.87 Å was obtained. This value takes into account, additively, all the differences between the overall docked complexes (inhibitor-enzyme), not only the poses of **Compound 1**(superimposed in S2 Fig).

#### Computational analysis

**Fig 10:**
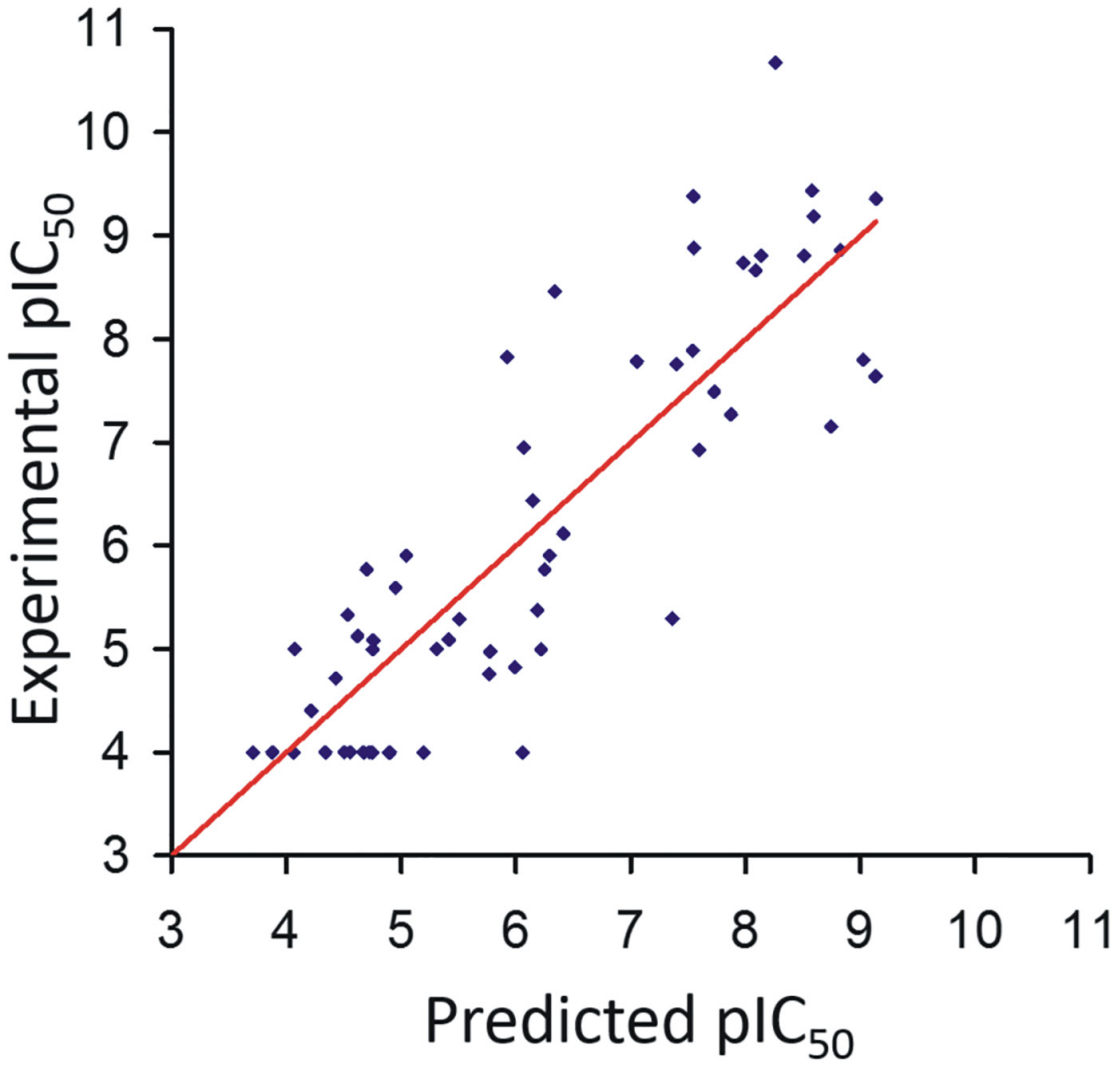
Graphic correlation of pIC_50_s, experimental data and values predicted via Equation [1]. Scatter plot showing the correlation between experimental and predicted pIC_50_ values according to Equation [1]. For the inactive compounds, the arbitrary value of 4.0 is assigned as experimental data.

In order to develop predictive relationships for inhibition activities and physico-chemical descriptors of the inhibitors, the computed scores and a set of ligand-based descriptors (S1 Table) were exploited to derive correlative equations (details in Materials and Methods). This approach allowed us also to confirm the reliability of the modelled inhibitor-enzyme complex (S1 File). Out of 1525 equations generated, applying a step-wise linear regression approach, we found the best correlation and the highest statistical significance for Equation [1] (Fig 10).

$$pIC_{50}=-4.4987+0.1418IMPR+0.6736Lip-1.5021MLPInS_{3}$$

$$\left(n=60;r^{2}=0.74,SE=0.996;F=54.14;P=5.55e^{-16};PC=59.439\right)$$

**Equation [1] and statistical evaluation** *Impropers* (*IMPR*) is the number of improper or pyramidal angles, excluding those constrained by an aromatic ring, *Lipole* (*Lip*) is lipophilicity moment and *MLP*_*InS3*_ is the docking score that includes the hydrophilic/hydrophobic complementarity between inhibitor and enzyme, and is dependent on the cube of the distance between interacting atom pairs (details in Materials and Methods). Since the molecules investigated are peptides, the improper angles are the number of H-bond donor-acceptor atoms, with the exclusion of the protonated amines. *N*, is the number of observations (molecules); *r* ^*2*^, the multiple correlation coefficient; *q* ^*2*^, the predictive squared correlation coefficient (obtained by leave-one-out cross-validation); *SE*, the standard deviation of errors; F, Fisher’s statistic coefficient; P, the significance for the F-test; and PC, Amemiya’s prediction criterion. A graphic representation of correlation between experimental and Equation [1] derived data is shown in Fig 10. According to this equation, inhibition activity is positively influenced by the number of atoms that can accept or donate H-bonds, as evidenced by the extended H-bond network stabilizing the complexes. The activity is also increased by molecules with high lipole, meaning that good inhibitors have hydrophilic and lipophilic centers widely separated. Lastly, the negative contribution of the docking descriptor MLP_InS3_ emphasizes the key role played by complementary polar and apolar contacts in stabilization of the inhibitor-enzyme complex (confirmed by the visual inspection of the docking poses). The robustness of the correlative Equation [1] was confirmed by randomly splitting the whole dataset into 20 pairs of training and test sets (details in Materials and Methods). For the test sets, we obtained a mean r^2^ of 0.72 (±0.07), comparable to that of Equation [1] (0.74). For all 20 training sets, the mean r^2^ value was 0.75 (±0.03).

### Biochemical and cellular analyses

#### Co-precipitation of PmV-inhibitors complexes

**Fig 11:**
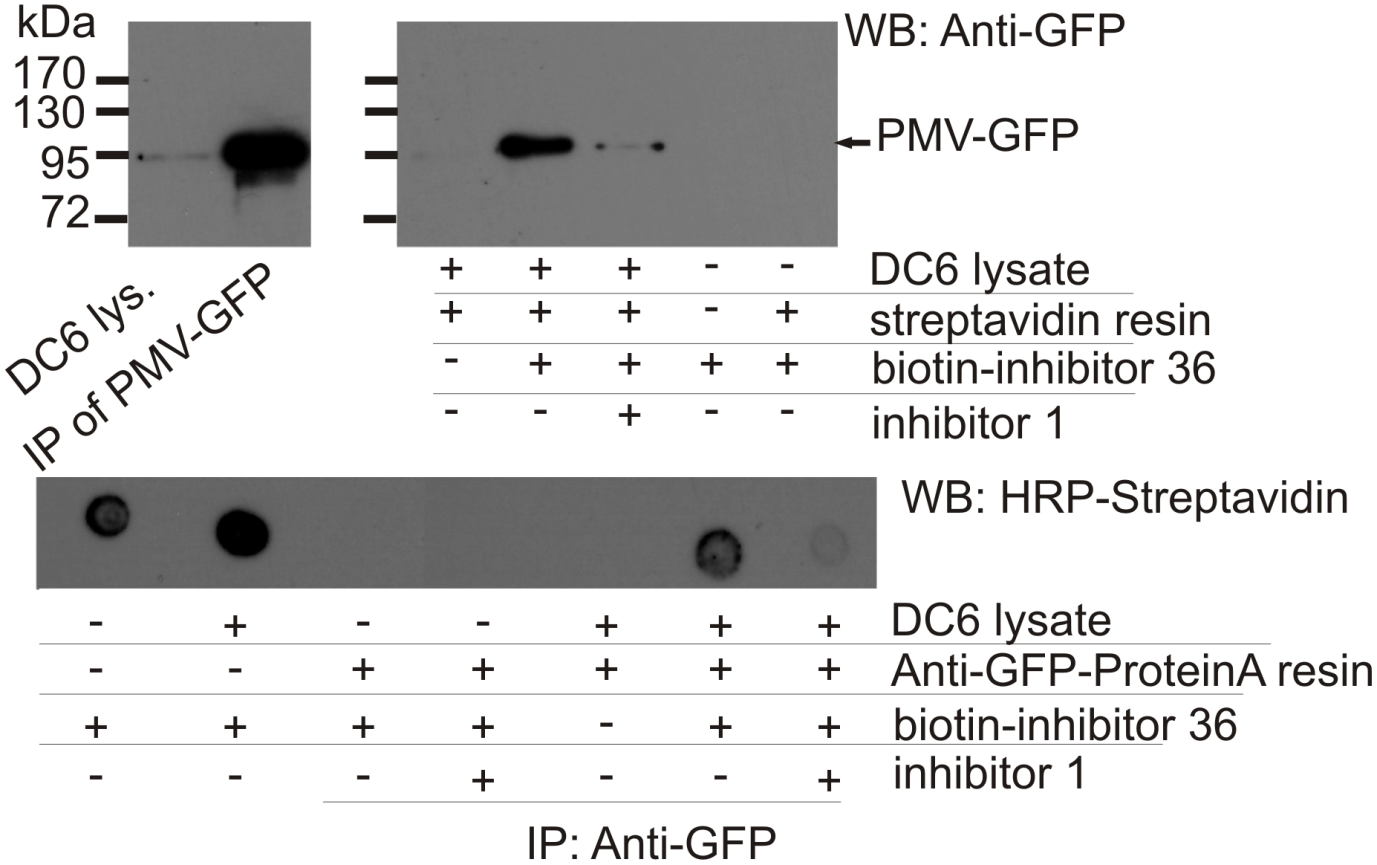
Interaction between PmV and Compound 36. **(A)** PmV detection after pulldown with **36**. **Compound 36** bound to streptavidin-resin was incubated with cellular lysates of DC6, that expresses PMV-GFP. Immuno-detection of GFP is shown. Controls: total cellular extract, immuno-precipitated PmV and resin not incubated with DC6 extracts. In the presence of **36**-streptavidin resin, PmV is pulled down, and this binding is inhibited by the presence of **Compound 1**. **(B)** Detection of **Compound 36** in immuno-precipitated PmV. After incubation of cell lysate with 50 μM **Compound 36** in the absence or presence of **Compound 1** (6^th^ and 7^th^ dots, respectively), PmV-GFP was immuno-precipitated with anti-GFP antibody 3E6. Biotin positivity is detected only in samples containing PmV (as shown in panel B, lane 2), indicating interaction between PmV and **36**. Similarly to panel B, **36**-binding is inhibited in the presence of **1**. Controls: **36** solution in presence and absence of DC6 cellular lysate (first two dots on the left), immuno-precipitation in absence of DC6 lysate (3^rd^ and 4^th^ dots) and immuno-precipitation in the absence of **36** (5^th^ dot), are included.

In order to assess the cellular permeability of our molecules and their interaction with PmV, we created **Compound 36**, a biotinylated version of **Compound 1**, that showed an *in vitro* inhibitory activity of ~ 42 nM (Fig 6 panel b). Complementary pull-down experiments using crude parasite lysates of clone DC6 [15] (that constitutively expresses PmV-GFP) in the presence or absence of **Compound 36**, confirmed the interaction between our inhibitor and PmV (Fig 11a and 11b). **Compound 36** was detected in association with immuno-precipitated PmV (Fig 11a). Conversely, PmV was detected after pull-down using **Compound 36** bound to streptavidin-resin (Fig 11b). We also show that **Compound 1**, acting as competitor, negatively affects the interaction between **Compound 36** and PmV (Fig 11a and 11b).

#### Cellular internalization of PmV-inhibitors

**Fig 12:**
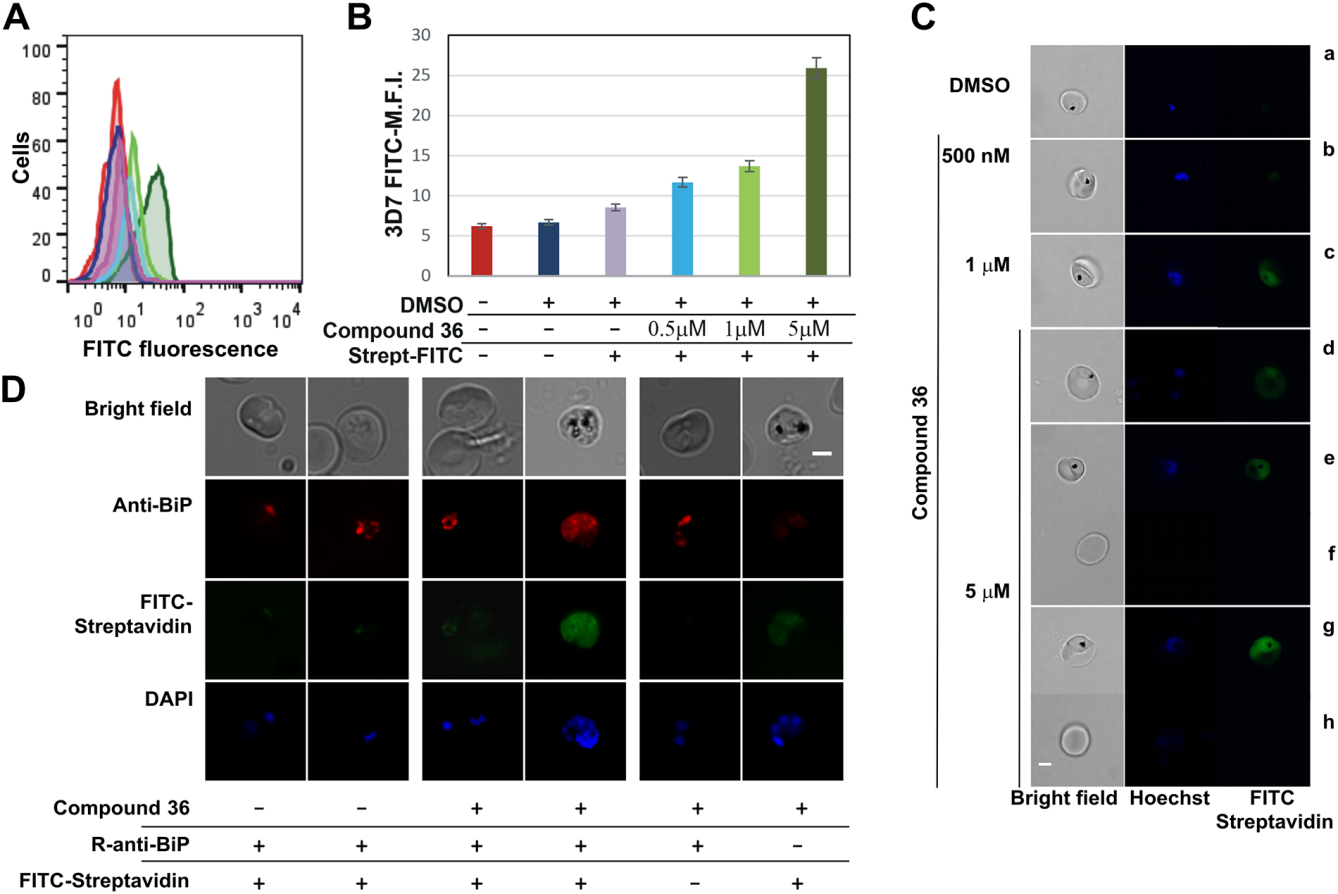
Analysis of Compound 36 internalization. **(A-B)** Flow cytometry analysis of **Compound 36**-treated cultures. Superimposed histograms (A) and mean fluorescence intensity (M.F.I.) graph (B) of the FITC channel (FL-1) of 3D7-parasitized cells are shown. The region corresponding to infected red blood cells is identified by nuclear staining with Propidium Iodide (0.5 μg/ml). The red line corresponds to untreated 3D7-parasites, blue, to DMSO-treated parasites; purple, to DMSO-treated parasites incubated with streptavidin-FITC; cyan, light and dark green, to **36**-treated parasites (at 500 nM, 1 μM and 5 μM, respectively). Bars represent ± Standard Deviation. **(C)** Microscopy analysis of **Compound 36** titration. After 6h incubation with **36** (panels b-h) at 500 nM (b), 1 μM (c) and 5 μM (d-h) or DMSO control (panel a), parasites were fixed, permeabilized and incubated with Streptavidin-FITC. Parasites are imaged in the presence of DAPI (nuclear staining). From left to right: bright field (in grey scale), Hoechst-stained nuclei (blue channel) and FITC-Streptavidin (green channel). Bar is 2 μm. The bottom two sets of panels (e-f and g-h) show comparison of fluorescence between infected and uninfected red blood cells in the same sample, slide and exposure times. All the images were acquired at a fixed exposure time and underwent equal contrast correction. **(D)** Microscopy analysis of **Compound 36** colocalization with BiP, an ER-marker. After incubation with **36** parasites were fixed, permeabilized and incubated with Streptavidin-FITC and/or rabbit-anti-BiP followed by the Alexa594-conjugated secondary antibody anti rabbit. Bright field (in grey scale), Alexa594/anti-BiP (red channel), Streptavidin-FITC (green channel) and DAPI (blue channel). Bar is 2 μm.

Using streptavidin-FITC binding, we also detected cellular internalization of **Compound 36**, with the degree of internalization correlating with the amount of inhibitor used (Fig 12). Importantly, **Compound 36** appears to be specifically internalized by parasites, as no significant streptavidin signal was detected in uninfected red blood cells, untreated parasitized cells or mock-treated parasite culture (Fig 12). Microscopy analyses of cellular distribution of **Compound 36** in relation to BiP marker of the endoplasmic reticulum [44] revealed that Biotinilated molecules are detected mostly in the cytoplasm and they are not excluded from the ER lumen. However, we have no means to ascertain the integrity of the internalized inhibitor. As the inhibitor comprises significant portions of peptidic backbone, it is possibly prone to degradation by other enzymes. No significant localization to the food vacuole was observed, in our fixation conditions [30].

#### Activity against parasite asexual growth

**Fig 13:**
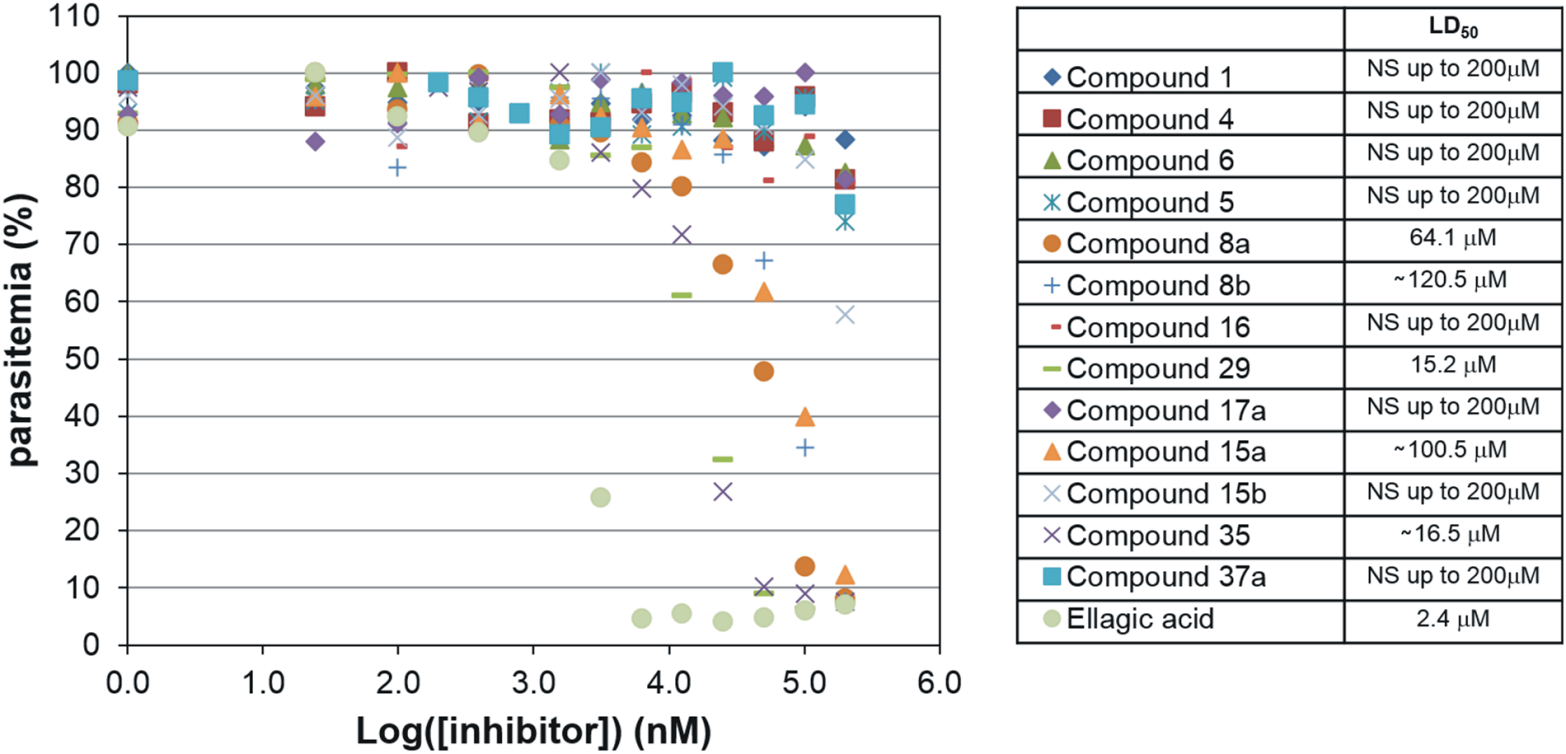
Growth inhibition. Growth inhibition curves of significant compounds are presented. Parasite cultures were exposed to titration curves of compounds for about 4 days. Parasitemia was evaluated via flow cytometry analysis using a nuclear staining (details in Material and Methods). Ellagic acid, known inhibitor of *Plasmodium* growth [45], was included as a positive control for the test. NS, Not significant.

Despite evidence of cellular internalization, all the synthetized compounds resulted in poor inhibition of parasite growth. The majority of the compounds did not show significant inhibition within the tested range of concentrations, 0.195–200 μM. Some of them gave measurable growth inhibition LD_50_s ranging from 10 to 150 μM (Fig 13). In these cases the treated cultures appear to die at the trophozoite stage as has been observed with other PmV inhibitors [20]. This may be due to the fact that, once inside the cell, our peptide-based inhibitors are either degraded or mis-localized, and/or that their lipophilic profiles do not guarantee an efficient access to PmV in the endoplasmic reticulum lumen. In fact, despite the detection of the internalization of **Compound 36** (Fig 12), the quantity and integrity of the internalized molecules could not be ascertained. The poor *in vivo* inhibition of the compounds did not allow a proper SAR. Interestingly, however, **Compounds 8a** and **15a**, missing one of the two charges of **Compound 1** (due respectively to the lack of the glutamate and the arginine), give measurable LD_50_s, while both their respective diastereoisomers, **8b** and **15b**, do not perform as well. **Compound 35**, carrying an acetyl group at the N-terminus, acts against parasite growth with a LD_50_ of 16.5 μM, while the best *in vitro* inhibitor of our series, **Compound 37a** (containing a more lipophilic N-terminus), up to 200 μM shows no significant activity against the parasites. More importantly, **Compound 29** exerted a reproducible lethal effect at ~ 15 μM, one of the lowest effective concentrations. **Compound 29**, containing a primary amine instead of the guanidinium in P_3_, showed *LogD*_pH7.4_ = −5.99, a better lipophilic profile than **Compound 1** (-4.28). **Compound 29** was selected to carry out *in vivo* validation of PmV as target for PExEl-based inhibitors in the following experiments.

#### In vivo inhibition of Plasmepsin V

**Fig 14:**
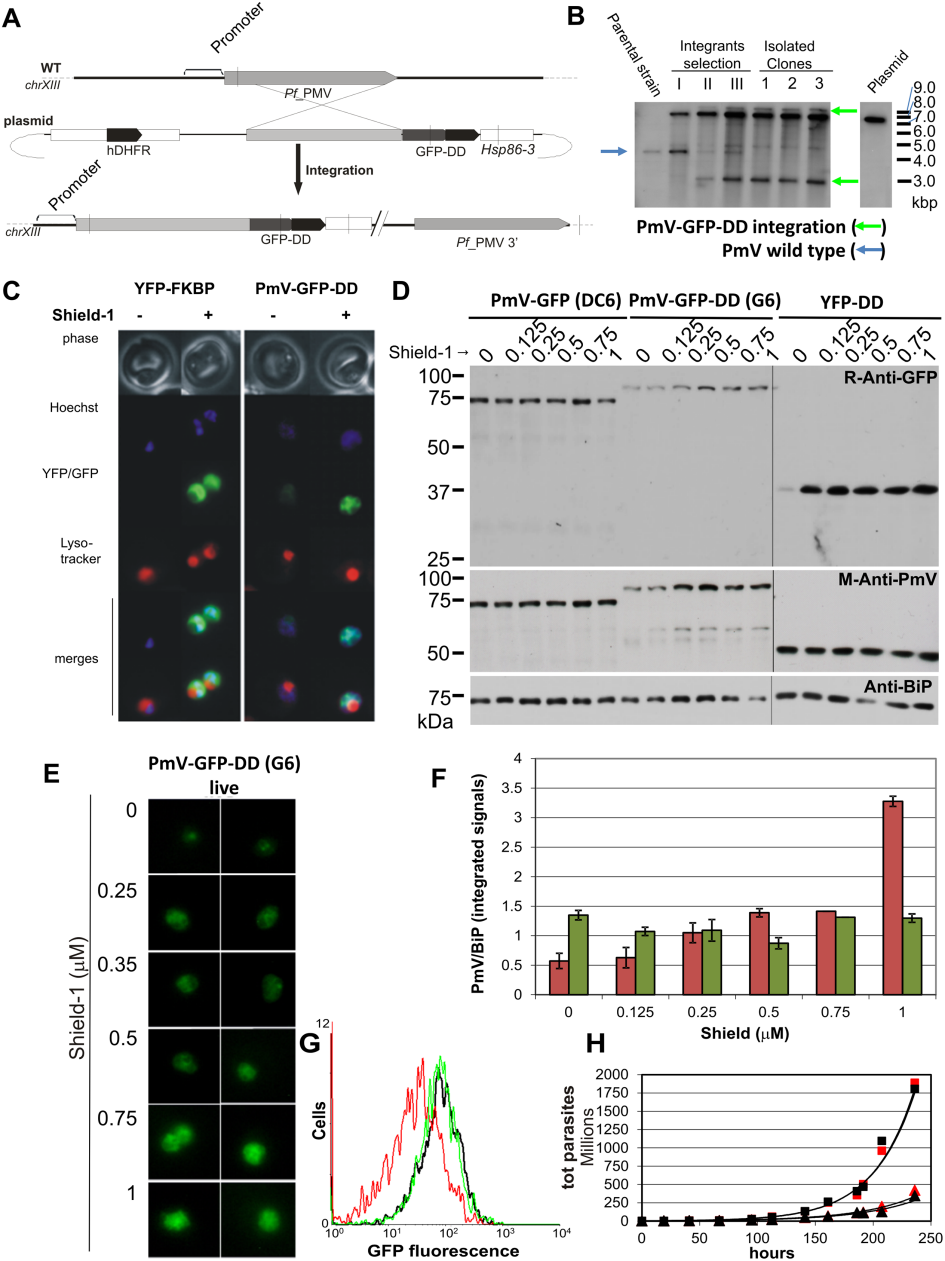
PmV-GFP-DD clone G6 generation and characterization. **(A)** Creation of a PmV-GFP-DD chimera by homologous recombination. The diagram shows the strategy to create C-terminally tagged PmV by integration at the endogenous locus. The plasmid contains sequence from the PmV ORF 3’ end in frame with GFP-DD [24]. Relative positions of BsrGI restriction sites (vertical bars) and the probe are indicated. **(B)** Southern blot of BsrGI-restricted DNA from the parental strain 3D7, the 3 drug cycles (Selection I, II, III), 3 of the isolated clones, among which G6 is number 3, and the transfected plasmid (at the far right) is shown. Arrows: endogenous gene (blue), and modified PmV locus (green). **(C)** Live microscopy of the clone expressing the cytosolic YFP-DD chimera (via episomally maintained plasmid) and G6 in presence or absence of 0.5 μM Shield-1. Top to Bottom: bright field (grey), Hoechst (blue), fluorescent proteins (green), Lysotracker (red), merges of the blue and green channels and of all three channels. **(D)** Western blot of total lysates of parasites at diverse concentrations of Shield-1 (0–1 μM): clone DC6, clone G6 and 3D7 expressing YFP-DD. PmV was detected with both anti-GFP and anti-PmV. BiP detection served as loading control. **(E)** Live microscopy of the G6 clone expressing the PmV-GFP-DD chimera at various Shield-1 concentrations (0–1 μM). **(F)** PmV levels from DC6 (green) and G6 (red) were quantified from blot (panel D) and plotted as ratio of PmV to BiP. The last point of the ratio is affected by a reduced BiP expression in 1 μM Shield-1. This is possibly due to the level of toxicity of this molecule at high concentrations. **(G)** Flow cytometry analysis of the GFP signal of PmV-GFP-DD (clone G6) in presence (green line) or absence (red line) of 0.75 μM Shield-1 in respect of PmV-GFP (clone DC6) (black line). The cellular levels of PmV-GFP(±DD) are similar in the presence 0.75 μM Shield-1 while PmV-GFP-DD is about 4 times less than PmV-GFP in the absence of Shield-1. **(H)** Growth curve analysis over 10 days for PmV-GFP-DD and DC6 in the presence or absence of 0.75 μM Shield-1. No significant effect on growth was observed in consequence of the reduced PmV levels.

**Fig 15:**
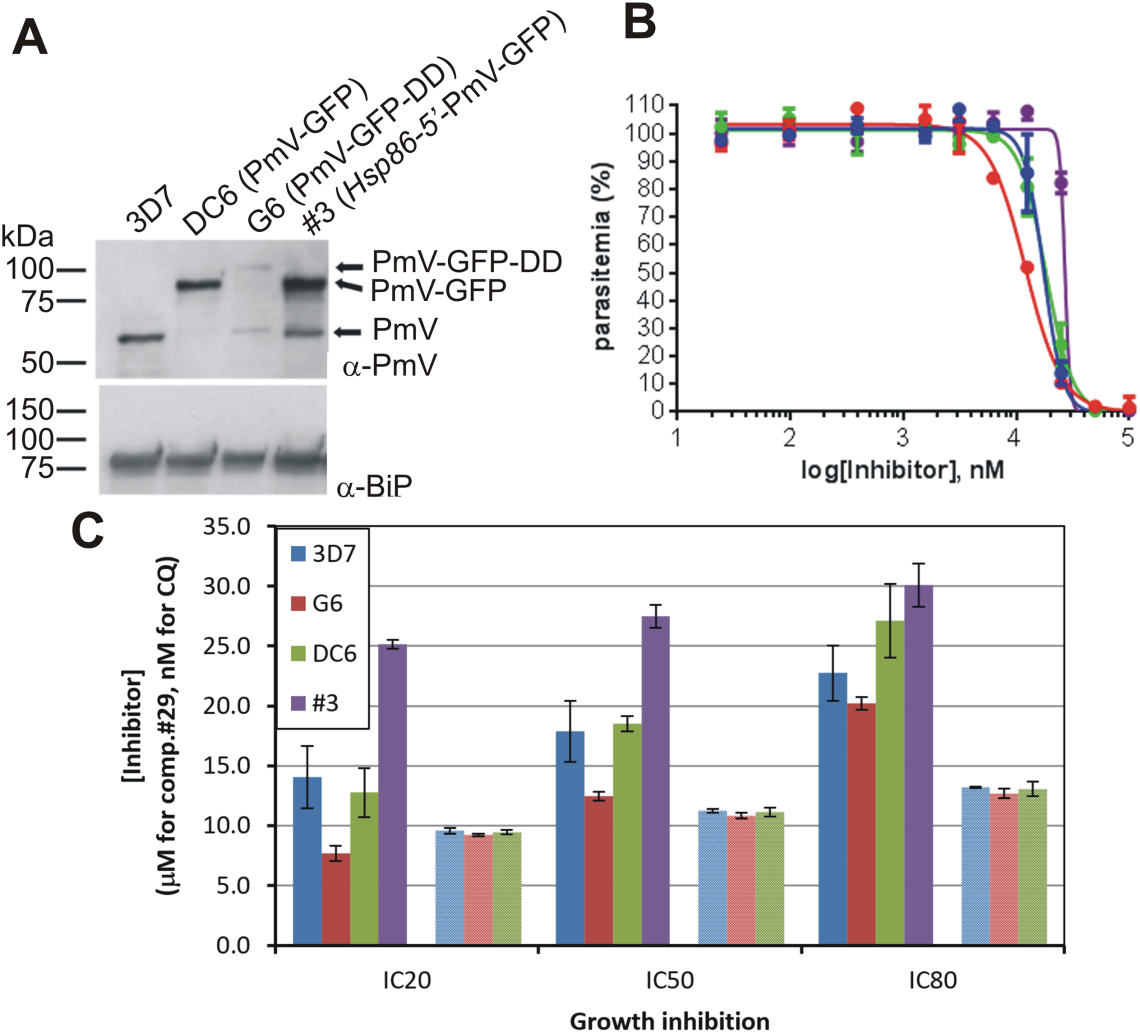
Inhibition correlates with cellular PmV levels. **(A)** Western blot analysis of cellular levels of PmV in the parental line, 3D7, and clonal parasites generated by genetic modifications. Immuno-detection of expressed PmV (upper panel) and BiP (lower panel), used as a loading control on the same SDS-PAGE gel, are shown. DC6 clone expresses PmV-GFP chimera under regulation of the native promoter [15]; G6 clone, the PmV-GFP-DD chimera under regulation of the native promoter (this paper) and #3 clone (episomally expressing PmV-GFP chimera under *Hsp86-5'*, a strong and constitutive promoter) [15]. G6 clone is maintained in absence of Shield-1. **(B)** Growth inhibition curves of parasites expressing different levels of PmV: 3D7 (blue dots and line), DC6 (green dots and line), G6 (PmV knock-down) (red dots and line) and #3 (Overexpressing PmV) (purple dots and line) were analysed in parallel. Growth inhibition was measured in triplicates. Dots represent actual data, lines the sigmoidal fitting obtained by non-linear regression analysis. **(C)** Shifts of inhibition curves detected by comparison of parasites expressing different levels of PmV. The sensitivities to **Compound 29** (bars with solid fill) and Chloroquine (CQ—bars with meshed fill) of 3D7, DC6, G6 (PmV knock-down) and #3 (Overexpressing PmV) were analysed in parallel. CQ sensitivity for #3 is was not determined. Comparisons of IC_20_, IC_50_ and IC_80_ values are shown in Table 1 and derived from curves shown in B. 3D7 and DC6 cultures expressed similar levels of Plasmepsin V; while G6 in the absence of Shield-1 showed decreased, and #3 showed augmented, levels of the cellular enzyme concentration. No significant shift were detected with CQ. Error bars show the standard deviation of the data.

A platform of cellular assays was set up in order to test, *in vivo*, the direct perturbation of both PmV activity and PExEl-secretion, using genetically modified parasites. Four different clones, all deriving from the parental strain 3D7, were used. These clones were: (a) HRPII-GFP, expressing a fluorescent probe for PExEl secretion under control of its native promoter [15]; and three clones producing different levels of PmV: (b) clone #3, expressing PmV-GFP, whose transcription is guided by a strong and constitutive promoter, *Hsp86-5'* (clone) [15]; (c) clone DC6, expressing PmV-GFP [15] and (d) clone G6 (this work) expressing PmV-GFP-DD, the latter two being under the control of the native PmV promoter. PmV-GFP-DD clone was generated by 3’ integration at the PmV locus of a plasmid carrying DNA encoding a GFP and a destabilization domain (DD) in frame to the PmV, as previously described [15, 23, 24], in order to generate an inducible knock-down (Fig 14a and 14b). The ‘destabilization domain’ (DD) derives from a FK506-binding, protein destabilization domain, which allows the regulation of protein levels [24, 28, 46]. PmV-GFP-DD clone G6 was genotyped by Southern Blot (clone number 3 in Fig 14b). The levels of PmV-knockdown were analysed in comparison to the episomal expression of a cytosolic YFP-DD and the effect on DC6 of the stabilizing drug Shield-1 by western blot (Fig 14d); live microscopy (Fig 14c and 14e); and flow cytometry (Fig 14g). PmV-GFP-DD yielded a reliable ~4–10 fold knockdown of PmV cellular levels (Figs 14f, 14g and 15a). In the absence of Shield-1, PmV-GFP-DD shows a GFP signal mostly localized in the food vacuole (Fig 14c and 14e) and a faint PmV-positive fragment at a molecular weight of about 60 kDa, that is possibly the result of a partial degradation (Fig 14d). The reduction of PmV we obtained does not seem to affect parasite viability to a detectable degree (Fig 14h), as previously observed using an alternative knock-down strategy [19]. We obtained for DC6 and G6, over a long course of growth in absence of Shield-1, growth constants (k) respectively, of 0.028 and 0.0274; doubling times (T) of 24.76 h and 25.3 h and a growth rate of (r) of 2.84 h^-1^ and 2.78 h^-1^ (derived from exponential fitting in Fig 14h). In the presence of 0.75 μM Shield-1, for DC6 and G6 the respective k values are 0.0199 and 0.0203; T values are 34.83 h and 34.15 h; and r values are 2.01 h^-1^ and 2.05 h^-1^ (Fig 14h).

**Table 1:** Inhibition concentrations of Compound 29 and Chloroquine in strains producing different levels of PmV.

**Table 1**						
**Compound 29**	**IC**_**20**_	SD	**IC**_**50**_	SD	**IC**_**80**_	SD
3D7	14.04	2.59	17.86	2.54	22.74	2.30
G6	7.68	0.62	12.45	0.36	20.22	0.53
DC6	12.77	2.04	18.50	0.64	27.13	3.08
#3	25.14	0.37	27.50	0.97	30.08	1.80
**Chloroquine**	**IC**_**20**_	SD	**IC**_**50**_	SD	**IC**_**80**_	SD
3D7	9.58	0.24	11.23	0.16	13.194	0.05
G6	9.23	0.11	10.83	0.23	12.70	0.38
DC6	9.45	0.19	11.11	0.37	13.06	0.61

Values of inhibition concentrations are calculated from sigmoidal fitting of the inhibition curves obtained in triplicates for cultures of different strains: the parental line, 3D7; clonal parasites: DC6 clone expressing the PmV-GFP chimera under the native promoter [15]; G6 clone, expressing a knockdown of PmV and #3 clone expressing the PmV-GFP chimera under *Hsp86-5'*, a strong and constitutive promoter [15].

Clone #3 overexpresses PmV-GFP up to 3–4 times more than the parental strain (Fig 15a). By measuring sensitivity to **Compound 29** in these cultures, we detected that growth inhibition curves are significantly shifted in clones G6 and #3, compared to 3D7, the parental strain, and the DC6, directly correlating with their respectively low and high PmV-cellular levels (Fig 15b and 15c). Shifts were more significant at lower dosages, IC_20_-IC_30_, than at higher concentrations (≥15 μM), at which indirect effects can possibly intervene, causing a less pronounced correlation with intracellular PmV levels (Fig 15b and 15c). No significant differences were observed between 3D7 and DC6 parasites in their sensitivity to **Compound 29** (Fig 15b and 15c) or to Chloroquine, a PmV-unrelated antimalarial drug. Values of inhibition concentrations are shown in Table 1.

#### In vivo inhibition of PExEl export

In consequence of the inhibition of PmV-dependent PExEl cleavage, the downstream *in vivo* effect should be the inhibition of the export of PExEl proteins. We, therefore, employed a parasite clone that constitutively expresses HRPII-GFP, as probe for PExEl-secretion in live cultures [15].

**Fig 16:**
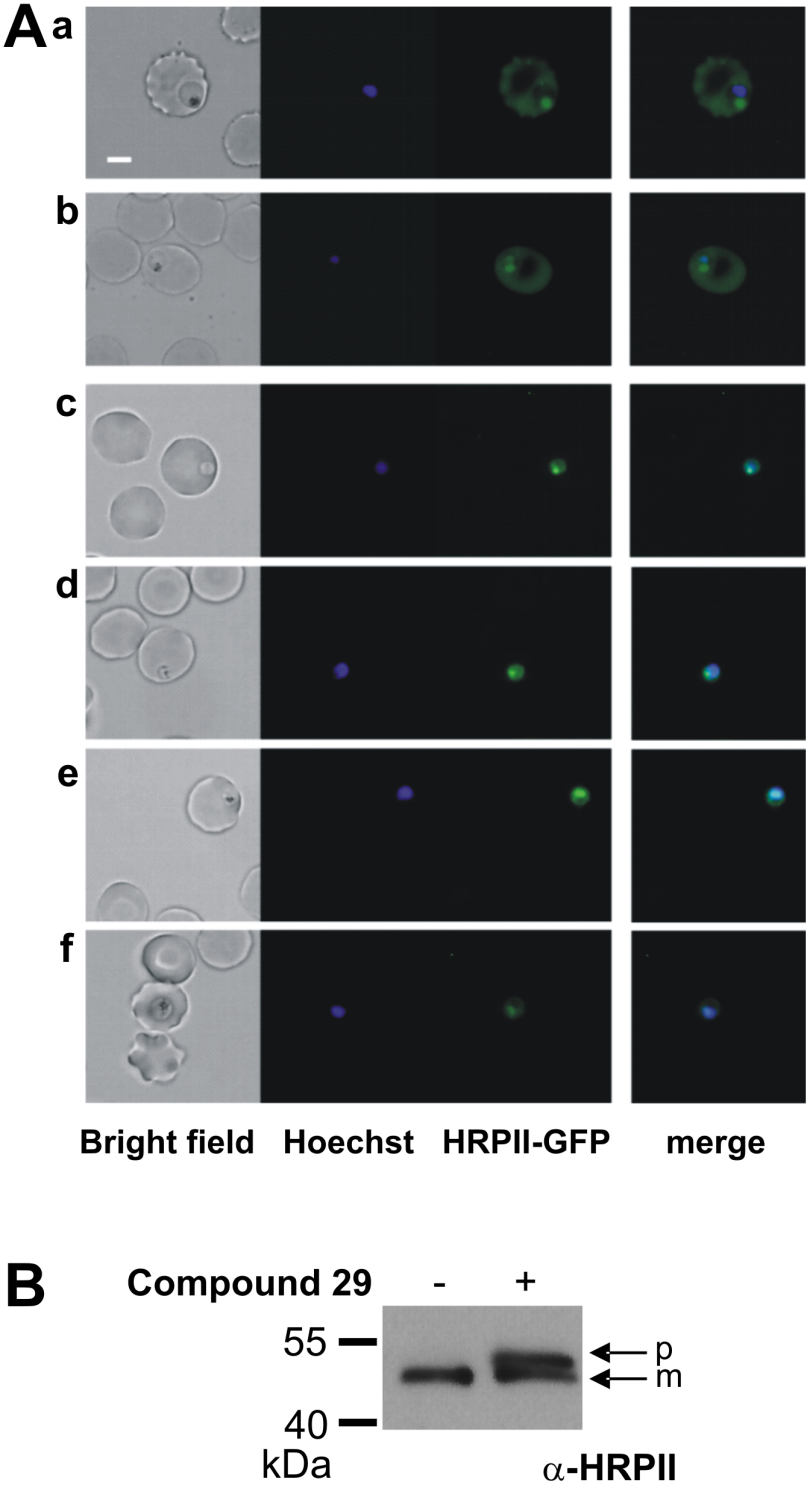
Inhibition results in impairment of the PExEl secretion. **(A)** Microscopy analysis of the distribution of the PExEl-export fluorescent probe. Live parasites expressing HRPII-GFP, used as a probe for PExEl secretion [15], are imaged in the presence of Hoechst nuclear staining. Bright field (grey scale), nuclei (blue channel), HRPII-GFP (green) and the merged image of the fluorescent channels are presented from left to right. (panel a) untreated culture; (b) mock DMSO-treated culture; (c-f) culture treated with 20 μM **29** solubilized in DMSO (c) mid ring, (d) late-ring, (e) early-trophozoite, (f) late-trophozoite. Bar is 2 μm. Impairment of HRPII-GFP PExEl-dependent export is observed in these representative images of live parasites and the GFP signal appears concentrated in regions of immediate proximity to the nuclei. In contrast, no export defects were observed in either the control or the mock DMSO-treated cultures. (B) HRPII PExEl processing is inhibited by **Compound 29**. Western blot analysis of HRPII contained in parasites after saponin treatment. Arrows show the pro-form (p) and the mature (m) protein HRPII. 3D7 culture was incubated as described in Materials and Methods with 20 μM **Compound 29** for about 27 h.

Upon treatment with **Compound 29**, an impairment of HRPII-GFP PExEl-dependent secretion was observed, as shown in representative images of live parasites, relative to control and mock-treated cultures (Fig 16a). Older parasites at the start of the treatment already showed, and some retained during the incubation, high levels of exported fluorescent protein, generated prior to exposure to inhibitors. Therefore, in order to detect defects in the PExEl-dependent export, we analysed parasites from ring to trophozoite stages. In these stages the fluorescent chimera in the presence of the inhibitor is mainly localized adjacent to the nuclei, resembling the secretion blocking effect of BrefeldinA. The natural processing of HRPII, due to Plasmepsin V, was shown to be inhibited by **Compound 29** (Fig 16b). Later-stage parasites seem to partially cope with PmV inhibition at the concentration employed (20 μM), as the GFP fluorescent probe, starting from late trophozoite stage, is detected in RBC cytosol. Similar observations have been previously reported for other PmV inhibitors [19].

## Discussion

PExEl-dependent secretion is a protein trafficking pathway recently discovered in *P*. *falciparum* [5, 6]. The novelty and considerable potential of this cellular process as an antimalarial target is currently attracting great interest [5, 6, 10, 11, 47]. Plasmepsin V (PmV), which cleaves the PExEl motif, is the key enzyme of this novel trafficking system [14, 15]. Only when it is cleaved, does the PExEl-motif target parasite proteins for secretion [48]. PmV is highly conserved in *Plasmodium* species [49]. It defines a new sub-class in the family of aspartic proteases [15, 16] and possesses only a distant homology to human beta-secretase (hBACE), which suggests a low probability of undesired side-effects for drugs targeting PmV [7, 15]. The high potential of PmV as drug target [18, 50] is supported by its refractoriness to genetic ablation [7, 9, 15, 17, 48] and by an expression profile spanning the entire *Plasmodium* life cycle [51–53]. However, despite its potential, this enzyme still lacks extensive enzymatic characterization and the identification of highly potent inhibitors. This work partly redresses these issues, providing new and efficient strategies for PmV inhibition.

We generated novel inhibitors for PmV by targeting the enzyme transition state, a methodology widely used for aspartic proteases [41, 54]. We obtained exceptionally potent inhibitors, with activity down to picomolar concentrations, by using the hydroxyethylamine (HEA) moiety. In comparison, similar inhibitors of other aspartic proteases, or when statine was employed as a transition-state mimic, have only achieved IC_50_s in the nanomolar range [19, 55]. **Compound 1**, one of the most potent inhibitors so far generated, closely resembles the PExEl-substrate but for the HEA moiety (S1 Fig); as such its initial binding affinity to the catalytic site (S_1-3_ and S_2_') would be expected to be similar to that of the natural substrate. However, the increased affinity of **Compound 1** over that of the natural substrate may depend on an efficient energetic stabilization of the transition state (likely to be via a disfavored dissociation constant). PExEl-dependent secretion is hindered as a result of PmV inhibition, and parasite growth impairment directly reflects intracellular levels of PmV [15, 19]. The PExEl-based inhibitors, described here, are able to target PmV *in vivo* and *in vitro*, inhibiting both PExEl-dependent secretion and parasite growth. Our results indicate that hydroxyethylamine, employed as a transition state mimic, is a novel and successful strategy for the inhibition of PmV.

There are no structural data for *P*. *falciparum* PmV currently available and only recently *P*. *vivax* PmV structure became available, while this paper was under revision [39]. Therefore, we were reliant solely on 3D models. The high variability of Pf_PmV 3D models obtained at the start of this work, suggested that reliance on predictive models was of limited utility in the initial design of inhibitors. With this observation in mind, we applied our inhibition strategy as a tool to explore the accessibility of the PmV catalytic site. By using a flexible workflow for the chemical synthesis (a protocol optimized from [56, 57]) rational modifications of the P and P' regions were generated, and used to perform SAR analysis of the requirements of PmV inhibition. The majority of our molecules efficiently inhibit PmV over a wide range of active concentrations. These include molecules which carry prohibitive modifications in respect to substrate requirements, such as R_1_ → K or R_1_ → W [7]. Surprisingly, these molecules inhibit PmV at low micromolar concentrations, performing better than previously reported HIV-inhibitors and Pepstatin A [14, 15].

Arginine in the first and leucine in the third position of the PExEl motif, are essential for substrate recognition, and are important for binding to the free enzyme [7, 19]. Whilst in the transition state conformation, S_3_ appears more permissive and the P_3_ arginine less indispensable. This observation is suggestive of significant conformational differences between PmV resting and transition states, as previously reported for other aspartic proteases [58], and this may significantly change requirements for efficient inhibition. Our observations and conclusions differ to those derived from use of the WEHI916 scaffold, which indicate the indispensability of the guanidinium group. This may be due to WEHI916 resembling only the P region of the PExEl. In contrast, our inhibitors may, by binding to both the S and the S' pockets of PmV, compensate for the loss of the optimal P_3_ interaction. Nevertheless, our results confirm that a stable, bulky and widely distributed positive charge in P_3_, as provided by guanidinium, is ideal for docking to the PmV active site. The modelling studies confirmed these observations by revealing a large electronegative S_3_ pocket, ideally coordinating the guanidinium group, but potentially exploitable by other electropositive moieties.

Our analysis suggests that interactions with the S_2_ pocket appear to be dispensable and only minimally contributory to inhibitory activity. Similar observations on P_2_ were reported in the study of Sleeb *et al*. using modifications of WEHI916 [20]. In conclusion, a branched aliphatic moiety of 2–4 carbons in P_2_ appears ideal in order to exploit the preferred hydrophobic profiles of S_2_ (our data and [20]). However, we show that S_2_ pocket tolerates slight polar perturbations, (**Compound 12)** and anticipate that a further exploration of this pocket may provide new insights into improved binding and possibly selectivity, as has been achieved for hBACE [59, 60].

The furthest C-terminus region does not appear to influence inhibition. Towards the N-terminus, we detected the presence of a putative hydrophobic pocket that accommodates aliphatic moieties, such as octanoic acid. The areas surrounding S_3_ have also been favorably exploited by the Cowman group, using a benzyl group upstream to the P_3_ [7]. However, it did not provide any greater activity *in vivo*.

WEHI916, a previously reported PmV inhibitor [19], inhibits PmV at 19–20 nM in an assay format significantly different from ours, presenting a different K_M_ value for the substrate employed (~ 9.7 μM) [19, 20]. They used an alternative substrate at half of the concentration we use (1.5 μM), a different buffer (including total ionic force and counter ions, all critical elements for aspartic protease mechanism [41]) and length of assay. Therefore, we directly tested WEHI916 in our assay. Its nanomolar inhibitory activity was confirmed, resulting in IC_50_ of 32.43 nM, ~ 1.7 times higher than previously reported [19, 20]. This insignificant difference is probably caused by the differing assay formats. Of greater importance is the side-by-side comparison of our **Compound 1** and WEHI916, which yielded a relative difference of ~ 100 fold greater inhibition efficiency in favor of **Compound 1** (Fig 3). The size difference of the two compounds is small (MWs of 664 Da for WEHI916 and 682 Da for **Compound 1**), while the main differences between these two molecules are the type of transition state mimic and the substrate regions involved in binding. **Compound 1** carries a hydroxyethylamine group while WEHI916 a statine. Also, very importantly, **Compound 1** interacts with both, S_1-3_ and S_1-3_', regions, whereas WEHI916 mainly binds to the S_1-4_ region. Our analysis of the shorter version of **Compound 1** helps to partially evaluate the importance of this second differing element for the inhibitory activity. Our **Compound 28a** (Fig 6 panel a) is the closest in size and homology to WEHI916, and interacts with S_1-3_ only. The fact that **28a** dramatically loses its activity in respect to its cognate, **Compound 7a**, indicates the importance of interactions with S_2_' for our longer compounds. However, **Compound 28a** also performs 100 times less efficiently than WEHI916, possibly because it lacks hydrophobic groups that extend the N-terminus of the peptidic backbone and interact with pre-S_3_ areas. The interaction with the hydrophobic pre—S_3_ region is very important, as confirmed by our **Compound 37a** (Fig 6 panel b). A shorter version of **37a**, lacking the residues P_2_' and P_3_' (similar to **Compound 28a**) would interact with S_1-3_ and the lipophilic pre-S_3_ regions, as WEHI916 does. Extrapolating from the data produced by **Compound 28a** relative to **Compound 7a**, an hypothetical **37a**_**ΔP2’-P3’**_ should yield a similar decrease in activity of ~ 2,000-fold. In fact, merely assuming that there are only additive effects on the variations of ΔG_binding_ (ΔG_b_) for such distant modifications, the shorter **37a**_**ΔP2’-P3’**_ would result in a ΔG_b_ of ~ -44 kJ mol^-1^ (as ΔG_b(7a)_− ΔG_b(28a)_ = -19.80 kJ mol^-1^) and in a relative IC_50_ of 26.38 nM, very close to WEHI916’s value. Despite the speculative nature of this analysis, our data suggests that the binding opportunities offered by the S' region and the lipophilic areas in close proximity to S_3_ are both critically important for potent inhibition of PmV, and that their exploitation should be pursued to further improve inhibitors.

The inhibitory activities of our compounds are favored by the contributions of interactions to the S' region. In this region, crucially important ionic interactions were detected in the pocket corresponding to the PExEl binding site S_2_'. Here, a distal carboxyl moiety appears to be required for maximum binding (Figs 5 and 6). In addition, inhibition activity is significantly reduced in the absence of P_2_' (**Compound 28a** versus **7a**), one of the less conserved positions of the PExEl-motif; but it is restored by the introduction of a glycine (**Compound 28a** versus **27a)** (Fig 6). Therefore, a hydrogen acceptor group, such as the glycine carboxyl group, plays an unexpectedly key role in binding with PmV S_2_'. Our docking analysis suggests that this interaction is mediated by Lys489 (Figs 7 and 8). Considering the results obtained from both the P_2_' modifications and the shorter inhibitors, this position clearly plays an important and previously unpredicted role in the stabilization of binding to the PmV transition state. In addition, if the interactions to the S' region are preserved, inhibition is achievable even when the binding to the S region is sub-optimal in low micromolar to high nanomolar ranges: for example, when arginine in P_3_ is switched to a lysine or even to aromatic amino acids (Figs 5 and 6). This evidence suggests the design of novel inhibitors lacking the highly hydrophilic and charged guanidinium group can still target PmV by stabilizing its transition state via maximized interactions in the S' region.

Since a clear correspondence with the previously published model was not found [61], even at the level of primary sequence and the Pv_PmV structure became available only few weeks ago [39], we based our docking analysis on our predictive model (S1 File). Despite its limitations as a basis for the *de novo* design of inhibitors, the docking followed by molecular dynamics in combination with the experimentally generated data yielded a reasonable estimation and validation of the possible Pf_PmV catalytic domain. The comparison of our model (S1 File) to the hypothetical structure obtained by employing as a template the recently published 3D structure of Pv_PmV [39] (S4 File) confirmed that our combined *in silico* and empiric methodology resulted in a useful approximation of the catalytic site conformation of plasmepsin V in *Plasmodia*. Our model was also challenged by computational analysis aiming to find reasonable physico-chemical descriptors for the detected inhibitory activities. We identified the best descriptors for PmV inhibitors as being the number of pyramidal angles; the lipophilic moment; and the docking score in S_3_. The validity of the derived equation was challenged by randomly feeding into the calculation ‘incorrect molecules’ and IC_50_ values, that were promptly identified as not fitting the data (data not shown). Our model robustly fits the experimentally obtained data and offers a starting platform for designing new non-peptidic scaffolds with high inhibitory potential. Nevertheless, we believe that the resolution of *P*. *falciparum* PmV 3D structure remains of crucial importance for future inhibitor design.

Interestingly, we noticed significant overall correspondences between the binding pockets of PmV and renin, which is responsible for angiotensinogen cleavage [62]. Both enzymes present a permissive S_2_ pocket for hydrophobic and slightly polar residues; and interactions with the prime site are critical for the efficient binding to the P_1_-P_3_ portion. These features may contribute significantly to the achievement of inhibitor picomolar affinity [63, 64]. In both of these proteases, the S_3_' site does not play an important role. In close proximity to the S_3_ there is a non-substrate hydrophobic pocket exploitable to enhance inhibition (this work and [20, 65]). In contrast, the major difference between the PmV and renin binding pockets is in the S_3_ pocket, which is unequivocally hydrophobic for renin, while hydrophilic for PmV, where P_3_ arginine is preferred ([7, 15, 20] and the present study). The exploitation of compound libraries previously evolved for renin inhibition would be an economical strategy to generate inhibitors to target PmV.

The peptidic inhibitors described here inhibit parasite growth with an LD_50_ not lower than the micromolar range, possibly due to poor membrane permeability. This is confirmed by their negligible access into the intracellular space of healthy erythrocytes. Unexpectedly, parasitized RBCs seem to acquire the biotinylated **Compound 36** molecule, possibly by utilizing alternative permeation routes generated by the parasite. This is an important observation concerning PExEl-mimetic compounds that may be of use for further pharmacokinetic investigation. The lipophilic profile of compounds is, however, critically important in ensuring access to the cellular location of PmV. Indeed, inhibitors are required to cross up to 4 membranes before reaching the endoplasmic lumen. Biotin was detected within the parasite at concentrations of 0.5–5 μM, much lower than those producing detectable growth impairment, at around 10–200 μM. It is probable, therefore, that our inhibitors are also affected by degradation (or modification) by parasite enzymes, and consequently only attain at high dosages PmV-inhibiting concentrations within the endoplasmic compartment. The recently published WEHI-916, while having a better lipophilic profile than our compounds, is similarly affected by poor and delayed accessibility to the endoplasmic reticulum lumen [19].

Previous inhibition strategies have focused on the first and the third PExEl-residues, the ones most highly conserved. Our main findings, that the arginine in P_3_ is dispensable for inhibition (as long as the interactions to the P' region are maximized) and that the pre-S_3_ lipophilic areas are of considerable importance for binding, will substantially aid the design of novel classes of PmV inhibitors. Future inhibitors, we envisage, will target the enzyme by exploiting these new potential binding possibilities in order to maximize potency and improve membrane permeation characteristics.

## Supporting Information

S1 Fig Schematics of the chemical synthesis of PmV inhibitors. Reagents and conditions: (a) to the resin is added a solution of bromomethylketone (150 μmol) [66] in DMF containing 150 μmol of DIEA. After stirring over-night the suspension is washed 6 times with DMF. (b) To the resin is added a solution, in DCM (2 mL), of Boc_2_O (750 μmol) and DIEA (1.15 mmol). The suspension is stirred for 1 h, filtered and washed 6 times with DCM. (c) The resin is suspended in a 1:1 mixture of THF/EtOH and 10 mg of NaBH_4_ is added. The mixture is stirred for 4 hours, filtered, washed 3 times with THF, 6 times with a 1:1 mixture of THF/H2O, twice with THF and finally 3 times with MeOH. (d) Cleavage: TFA, TIS, thioanisole, phenol, followed by precipitation (MTBE) and preparative HPLC. Abbreviations: SPPS, solid phase peptide synthesis; DMF, dimethylformamide; Fmoc, fluorenylmethyloxycarbonyl; BOC, *tert*-butoxycarbonyl; DIEA, N,N-diisopropylethylamine; DCM, dichloromethane; THF, tetrahydrofuran; TFA, trifluoroacetic acid; TIS, triisopropylsilane; MTBE, methyl *tert*-butyl ether.
(PDF)

S2 Fig Superimposition of the two Pf_PmV 3D models presented in this paper. Superimposition of the Pf_PmV 3D-model used in this work (blue ribbon) (PDB file in S1 File) and the one derived from modelling Pf_PmV to the recently published structure of Pv_PmV, in complex with WEHI-842 (red ribbon) [39] (PDB file in S3 File) was done by aligning the alpha carbons of the peptidic backbones. 3D models of PmV were obtained by Phyre 2 homology modelling software using as templates either pro-plasmepsin of *Plasmodium vivax* (PDB code 1MIQ) or the recently published Pv_PmV (PDB code 4ZL4) (red ribbon) [39]. The two catalytic aspartates and the β-sheet flap, that form the aspartic protease catalytic groove, are indicated by yellow arrows.
(PDF)

S1 File Pf_PmV 3D model obtained using pro-plasmepsin of *Plasmodium vivax* (PDB code 1MIQ) as template. The 3D model was obtained by Phyre 2 homology modelling software
(PDB)

S2 File Animation of the *in-silico* 3D model of PmV active site bound to *(S)*-1 presented in Fig 7. 3D representation of *(S)*-1-PmV complex was obtained by Phyre 2 homology modelling software. **Compound 1** is represented as solid ‘balls and sticks’ while PmV residues surrounding the catalytic pocket are as wireframe (C, N, O, H are respectively green, blue, red and white). Only hydrophilic hydrogens are included.
(AVI)

S3 File Alignment of *P*. *falciparum* Plasmepsin V (gene code PF3D7_1323500) and *P*. *vivax* Plasmepsin V (gene code PVX_116695). The alignment of Pf_PmV and Pv_PmV was obtained by the software EMBOSS Needle from EMBL-EBI. “ǀ” indicates identity between the amino acids of the sequences in the upper and lower lines, while “.” indicates similarity.
(PDF)

S4 File Pf_PmV 3D model obtained using the recently published structure of Pv_PmV in complex with WEHI-842 [39] as template. The 3D model was obtained by Phyre 2 homology modelling software.
(PDB)

S1 Table Computed scores of the physico-chemical descriptors of the inhibitors and the ligand-based descriptors used in the computational analysis. (XLS)
