# Correction: Picomolar Inhibition of Plasmepsin V, an Essential Malaria Protease, Achieved Exploiting the Prime Region

**DOI:** 10.1371/journal.pone.0146627

**Published:** 2016-01-04

**Authors:** Luca Gambini, Luca Rizzi, Alessandro Pedretti, Orazio Taglialatela-Scafati, Mario Carucci, Andrea Pancotti, Corinna Galli, Martin Read, Emanuele Giurisato, Sergio Romeo, Ilaria Russo

The following information is missing from the Funding section: Work by IR and co-workers is primarily supported by Wellcome Trust Infrastructure Support Fund of the University of Manchester and the European Research Council.

There is an error in the Acknowledgments section. This section should read: We thank Jacobus Pharmaceutical Company, Princeton, NJ for the kind provision of WR99210 and ATCC (MR4) for BiP antibody, D. Taylor for HRPII antibody, L. Romani and A. Vecchiarelli for fluorimeter access; and B. Sleebs and J. Boddey for WEHI916.
