# Supplementary figures and images for: Picomolar Inhibition of Plasmepsin V, an Essential Malaria Protease, Achieved Exploiting the Prime Region
Source: PLoS One. 2015 Nov 13;10(11):e0142509. doi: 10.1371/journal.pone.0142509 (PMC4643876; doi:10.1371/journal.pone.0142509)

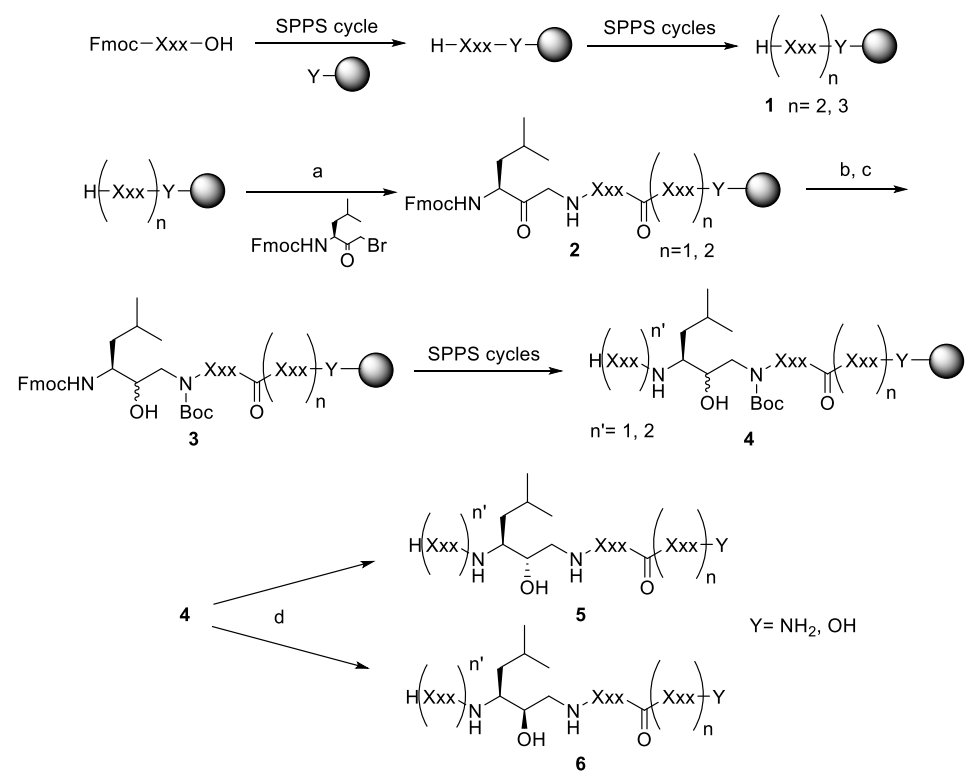

Supplement: S1 Fig — Reagents and conditions: (a) to the resin is added a solution of bromomethylketone (150 μmol) [66] in DMF containing 150 μmol of DIEA. After stirring over-night the suspension is washed 6 times with DMF. (b) To the resin is added a solution, in DCM (2 mL), of Boc2O (750 μmol) and DIEA (1.15 mmol). The suspension is stirred for 1 h, filtered and washed 6 times with DCM. (c) The resin is suspended in a 1:1 mixture of THF/EtOH and 10 mg of NaBH4 is added. The mixture is stirred for 4 hours, filtered, washed 3 times with THF, 6 times with a 1:1 mixture of THF/H2O, twice with THF and finally 3 times with MeOH. (d) Cleavage: TFA, TIS, thioanisole, phenol, followed by precipitation (MTBE) and preparative HPLC. Abbreviations: SPPS, solid phase peptide synthesis; DMF, dimethylformamide; Fmoc, fluorenylmethyloxycarbonyl; BOC, tert-butoxycarbonyl; DIEA, N,N-diisopropylethylamine; DCM, dichloromethane; THF, tetrahydrofuran; TFA, trifluoroacetic acid; TIS, triisopropylsilane; MTBE, methyl tert-butyl ether. (PDF) [file pone.0142509.s001.pdf]

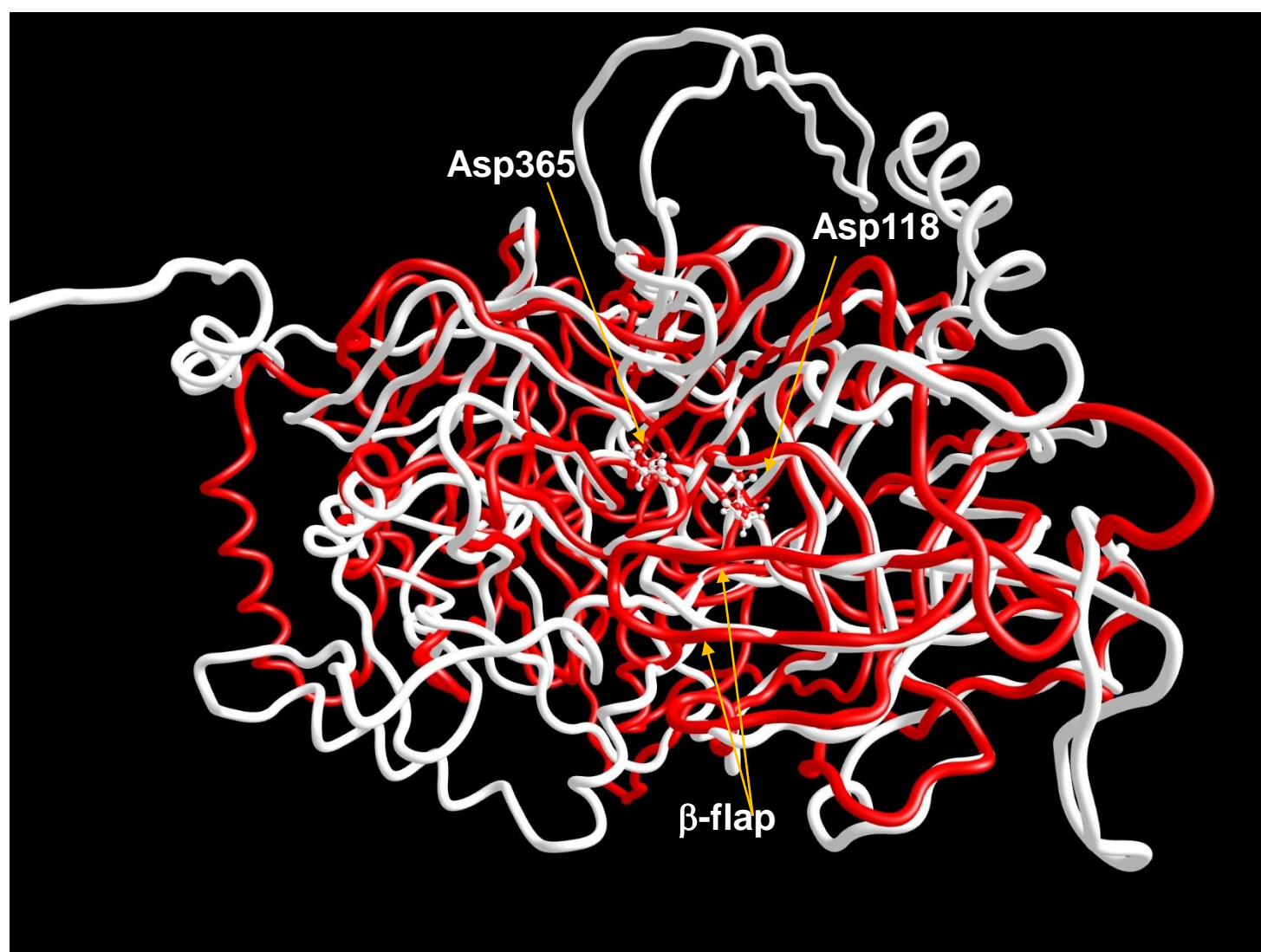

Supplement: S2 Fig — Superimposition of the Pf_PmV 3D-model used in this work (blue ribbon) (PDB file in S1 File) and the one derived from modelling Pf_PmV to the recently published structure of Pv_PmV, in complex with WEHI-842 (red ribbon) [39] (PDB file in S3 File) was done by aligning the alpha carbons of the peptidic backbones. 3D models of PmV were obtained by Phyre 2 homology modelling software using as templates either pro-plasmepsin of Plasmodium vivax (PDB code 1MIQ) or the recently published Pv_PmV (PDB code 4ZL4) (red ribbon) [39]. The two catalytic aspartates and the β-sheet flap, that form the aspartic protease catalytic groove, are indicated by yellow arrows. (PDF) [file pone.0142509.s002.pdf]
