# Supplementary material for: Picomolar Inhibition of Plasmepsin V, an Essential Malaria Protease, Achieved Exploiting the Prime Region
Source: PLoS One. 2015 Nov 13;10(11):e0142509. doi: 10.1371/journal.pone.0142509 (PMC4643876; doi:10.1371/journal.pone.0142509)
Supplement: S3 File — The alignment of Pf_PmV and Pv_PmV was obtained by the software EMBOSS Needle from EMBL-EBI. “ǀ” indicates identity between the amino acids of the sequences in the upper and lower lines, while “.” indicates similarity. (PDF) [file pone.0142509.s005.pdf]

```
########################################
# Program: needle
# Rundate: Thu  3 Sep 2015 14:59:12
# Commandline: needle
#    -auto
#    -stdout
#    -asequence emboss_needle-I20150903-145911-0687-75765244-oy.asequence
#    -bsequence emboss_needle-I20150903-145911-0687-75765244-oy.bsequence
#    -datafile EBLOSUM62
#    -gapopen 10.0
#    -gapextend 0.5
#    -endopen 10.0
#    -endextend 0.5
#    -aformat3 pair
#    -sprotein1
#    -sprotein2
# Align_format: pair
# Report_file: stdout
########################################

#=======================================
#
# Aligned_sequences: 2
# 1: PmV Vivax (Vivax)
# 2: PmV Falciparum (Falciparum)
# Matrix: EBLOSUM62
# Gap_penalty: 10.0
# Extend_penalty: 0.5
#
# Length: 614
# Identity:     323/614 (52.6%)
# Similarity:   418/614 (68.1%)
# Gaps:          94/614 (15.3%)
# Score: 1802.5
# 
#
#=======================================

Vivax              1 ------------------------------------------------MV      2
                                                                     .|
Falciparum         1 MNNYFLRKENFFILFCFVFVSIFFVSNVTIIKCNNVENKIDNVGKKIENV     50

Vivax              3 GASLGPPGRGSLSRLIRLVICVLTLCALSVQGRSESTEGHSKDL------     46
                     |..:|                       .::.::::.|..:.::      
Falciparum        51 GKKIG-----------------------DMENKNDNVENKNDNVGNKNDN     77

Vivax             47 -------LYKYKLYGDIDEYAYYFLDIDIGTPEQRISLILDTGSSSLSFP     89
                            |||||||||||||||||||||||.|.|||||||||||||||||
Falciparum        78 VKNASSDLYKYKLYGDIDEYAYYFLDIDIGKPSQRISLILDTGSSSLSFP    127

Vivax             90 CAGCKNCGVHMENPFNLNNSKTSSILYCENEECPFKLNCVKGKCEYMQSY    139
                     |.|||:||:|||.|:|||.|||||||||....||:.|.||..||||:|||
Falciparum       128 CNGCKDCGIHMEKPYNLNYSKTSSILYCNKSNCPYGLKCVGNKCEYLQSY    177

Vivax            140 CEGSQISGFYFSDVVSVVSYNNE-RVTFRKLMGCHMHEESLFLYQQATGV    188
                     ||||||.||||||:|::.||||: :::|.||||||||||||||:||||||
Falciparum       178 CEGSQIYGFYFSDIVTLPSYNNKNKISFEKLMGCHMHEESLFLHQQATGV    227

Vivax            189 LGMSLSKPQGIPTFVNLLFDNAPQLKQVFTICISENGGELIAGGYDPAYI    238
                     ||.||:||.|:||||:|||.:.|.||.:::||:||:|||||.|||:|.|.
Falciparum       228 LGFSLTKPNGVPTFVDLLFKHTPSLKPIYSICVSEHGGELIIGGYEPDYF    277
```

```
Vivax          239 VRRGGSKSVSESVSGQGSGPVSESLSESLSESGEDPQ------VALREAE 282
                   :.....|...:......|...:.|:....::..:|.:      :.....|
Falciparum     278 LSNQKEKQKMDKSDNNSSNKGNVSIKLKNNDKNDDEENNSKDVIVSNNVE 327

Vivax          283 KIVWENVTRKYYYYIKVRGLDMFGTNMMSSSKGLEMLVDSGSTFTHIPED 332
                   .|||:.:|||||||||:.|||::|||:| ..|.|:||||||||||||||:
Falciparum     328 DIVWQAITRKYYYYIKIYGLDLYGTNIM-DKKELDMLVDSGSTFTHIPEN 376

Vivax          333 LYNKLNYFFDILCIQDMNNAYDVNKRLKMTNESFNNPLVQFDDFRKSLKS 382
                   :||::||:.|||||.||.|.|::|||||:||||.|.|||.|:||:.:||:
Falciparum     377 IYNQINYYLDILCIHDMTNIYEINKRLKLTNESLNKPLVYFEDFKTALKN 426

Vivax          383 IIAKENMCVKIVDGVQCWKYLEGLPDLFVTLSNNYKMKWQPHSYLYKKES 432
                   ||..||:|:||||||||||.||.||:|::||||||||.|:|.||||||||
Falciparum     427 IIQNENLCIKIVDGVQCWKSLENLPNLYITLSNNYKMIWKPSSYLYKKES 476

Vivax          433 FWCKGIEKQVNNKPILGLTFFKNRQVIFDIQKNRIGFVDANCPSHPTHTR 482
                   |||||:|||||||||||||||||:|||||:|:|:|.|:::.|||:.|.:|
Falciparum     477 FWCKGLEKQVNNKPILGLTFFKNKQVIFDLQQNQIAFIESKCPSNLTSSR 526

Vivax          483 PRTYNEYKRKDNIFLKIPFFYLYSLFVVFALSVLLSLVFYVRRLYHMEYS 532
                   |||:|||:.|:|||||:.:..||.|:::.||::||||:.|||::::|:|.
Falciparum     527 PRTFNEYREKENIFLKVSYINLYCLWLLLALTILLSLILYVRKMFYMDYF 576

Vivax          533 PLPSEGKAPADA--    544
                   ||..:.|:|...
Falciparum     577 PLSDQNKSPIQEST    590

#---------------------------------------
#---------------------------------------
```
